# Adjusting expected deaths for mortality displacement during the COVID-19 pandemic: a model based counterfactual approach at the level of individuals

**DOI:** 10.1186/s12874-023-01984-8

**Published:** 2023-10-18

**Authors:** Richard James Holleyman, Sharmani Barnard, Clarissa Bauer-Staeb, Andrew Hughes, Samantha Dunn, Sebastian Fox, John N. Newton, Justine Fitzpatrick, Zachary Waller, David John Deehan, Andre Charlett, Celia L. Gregson, Rebecca Wilson, Paul Fryers, Peter Goldblatt, Paul Burton

**Affiliations:** 1 UK Health Security Agency, Wellington House; 133–155 Waterloo Road, London, SE1 8UG UK; 2 Population Health Sciences Institute, Newcastle University, Newcastle Upon Tyne, NE1 7RU UK; 3 School of Population Health, Curtin University, Bentley, WA 6102 Australia; 4 Office for Health Improvement and Disparities, Department of Health and Social Care, 39 Victoria Street, London, SW1H 0EU UK; 5 Newcastle Upon Tyne Hospitals NHS Foundation Trust, Freeman Road, High Heaton, Newcastle Upon Tyne, NE7 7DN UK; 6 Musculoskeletal Research Unit, Translational Health Sciences, Bristol Medical School, University of Bristol, Bristol, BS8 1QU UK; 7 Department of Public Health, Policy and Systems, University of Liverpool Waterhouse Building, Block B, Brownlow Street, Liverpool, L69 3GL UK; 8 Department of Epidemiology & Public Health, UCL Institute of Health Equity, University College London, 1-19 Torrington Place, London, WC1E 7HB UK

**Keywords:** Displacement, Harvesting, COVID-19, Counterfactual, Excess mortality, Excess death

## Abstract

**Background:**

Near-real time surveillance of excess mortality has been an essential tool during the COVID-19 pandemic. It remains critical for monitoring mortality as the pandemic wanes, to detect fluctuations in the death rate associated both with the longer-term impact of the pandemic (e.g. infection, containment measures and reduced service provision by the health and other systems) and the responses that followed (e.g. curtailment of containment measures, vaccination and the response of health and other systems to backlogs). Following the relaxing of social distancing regimes and reduction in the availability of testing, across many countries, it becomes critical to measure the impact of COVID-19 infection. However, prolonged periods of mortality in excess of the expected across entire populations has raised doubts over the validity of using unadjusted historic estimates of mortality to calculate the expected numbers of deaths that form the baseline for computing numbers of *excess* deaths because many individuals died earlier than they would otherwise have done: i*.e. their* mortality was displaced earlier in time to occur during the pandemic rather than when historic rates predicted. This is also often termed “harvesting” in the literature.

**Methods:**

We present a novel Cox-regression-based methodology using time-dependent covariates to estimate the profile of the increased risk of death across time in individuals who contracted COVID-19 among a population of hip fracture patients in England (*N* = 98,365). We use these hazards to simulate a distribution of survival times, in the presence of a COVID-19 positive test, and then calculate survival times based on hazard rates without a positive test and use the difference between the medians of these distributions to estimate the number of days a death has been displaced. This methodology is applied at the individual level, rather than the population level to provide a better understanding of the impact of a positive COVID-19 test on the mortality of groups with different vulnerabilities conferred by sociodemographic and health characteristics. Finally, we apply the mortality displacement estimates to adjust estimates of excess mortality using a “ball and urn” model.

**Results:**

Among the exemplar population we present an end-to-end application of our methodology to estimate the extent of mortality displacement. A greater proportion of older, male and frailer individuals were subject to significant displacement while the magnitude of displacement was higher in younger females and in individuals with lower frailty: groups who, in the absence of COVID-19, should have had a substantial life expectancy.

**Conclusion:**

Our results indicate that calculating the expected number of deaths following the first wave of the pandemic in England based solely on historical trends results in an overestimate, and excess mortality will therefore be underestimated*.* Our findings, using this exemplar dataset are conditional on having experienced a hip fracture, which is not generalisable to the general population. Fractures that impede mobility in the weeks that follow the accident/surgery considerably shorten life expectancy and are in themselves markers of significant frailty. It is therefore important to apply these novel methods to the general population, among whom we anticipate strong patterns in mortality displacement – both in its length and prevalence – by age, sex, frailty and types of comorbidities. This counterfactual method may also be used to investigate a wider range of disruptive population health events. This has important implications for public health monitoring and the interpretation of public health data in England and globally.

**Supplementary Information:**

The online version contains supplementary material available at 10.1186/s12874-023-01984-8.

## Introduction

During the COVID-19 pandemic near-real time surveillance of excess mortality has been an essential tool for detection of increased and unexpected mortality in many countries [1–5]. It is sensitive both to direct and indirect effects of the pandemic and is not dependent on COVID-19 testing patterns [6]. It provides information for managing the timing and extent of COVID-19 containment measures, planning for increased demands placed on health services, and remains critical for monitoring longer-term impacts of the pandemic on specific causes of mortality [7].

Excess mortality compares observed mortality with expected mortality, where expected mortality is commonly estimated using historic mortality rates. However, the pandemic has resulted in prolonged periods of mortality in excess of the number expected across the entire population. Without substantive periods of respite from waves of infection there has not been a ‘catch-up period’ long enough for deaths to return to the level expected based on pre-pandemic trends. Therefore, estimates of the expected number of deaths (and therefore excess deaths) beyond the first wave of the pandemic are increasingly unreliable for several challenging reasons, some of which we address in this paper:

1. Deaths due to COVID-19 occurred among individuals who would otherwise have been expected to live longer [8].
2. People with co-morbidities were more likely than others to die early following COVID-19 infection. Reported mortality rates from these causes (e.g., acute coronary syndrome) will be lower, both during and after the pandemic, than they would have been in the absence of the COVID-19 pandemic [9, 10].
3. Conversely, some elective surgery or other types of treatment for co-morbidities were postponed during the pandemic, reducing the number of deaths expected to have occurred as a consequence of the surgery during the pandemic. However, delays in treatment might lead to higher long-term mortality from the affected conditions [11–13].
4. Disruption to normal life, during what has proved to be a lengthy pandemic, is likely to have affected levels of mortality and made the use of historic rates less reliable in estimating expected numbers of deaths. This disruption includes mobility restrictions, suppression of non-COVID-19 infections such as influenza, on-going public reticence to engage with health services, and the unknown effects of surviving COVID-19 infection on long-term mortality risk [14, 15].

For reasons 1–2, estimates of expected mortality are distorted by substantive and sometimes complex ‘mortality displacement’ due to COVID-19. Establishing an accurate prediction of expected deaths is crucial for routine surveillance as well as monitoring the ongoing and longer-term impacts of the pandemic. In this paper we focus on the displacement of mortality experienced by those who had a positive COVID-19 test result.

To date, population level model-based approaches that aim to estimate short term displacement of mortality following an exogenous shock have typically involved the use of Poisson or Quasi-Poisson non-linear time-lag models [16, 17]. However, the extent to which an individual's mortality is displaced is dependent on their underlying risk of mortality prior to infection. Furthermore, in the COVID-19 pandemic, unlike a community-wide temperature shock in which the whole population is ‘at risk’, it is only once an individual has contracted COVID-19 that they suffer a markedly increased risk of their death being brought forward. Thus, an effective analysis must focus on the mortality of *individuals* in relation to rapid changes in their underlying risk profile with time and none of the previous methods naturally extend to encompass this possibility. Furthermore, the complex setting of repeated waves of COVID-19 infection affecting different individuals makes it difficult to apply any simple interpolation method.

In this paper, we use an exemplar dataset of patients from the English National Hip Fracture Database (NHFD) to adopt a Cox-regression-based methodology using time-dependent covariates to estimate the profile of the enhanced risk of death across time in individuals who contracted COVID-19 [18, 19]. This general approach can be applied to any adverse event-based outcome (death being just one example) that follows any ‘at-risk’ defining event (here it just happens to be developing COVID-19), to investigate impacts by time or by cause.

The hip fracture population represents an ideal cohort in which to study mortality displacement for several reasons. Firstly, hip fracture represents one of the most common serious injuries in older people following which the mortality rate is high [20], which we anticipate will produce relatively short mortality displacement estimate times falling within the follow-up time-period available for Cox modelling – i.e. a large number of individuals who sustained a hip fracture could be expected to have died during the available follow-up time, regardless of whether they contracted COVID-19. Secondly, most individuals sustaining a hip fracture will be treated as a hospital inpatient where the risk of nosocomial exposure to COVID-19 was high, and we can have greater confidence in accurately capturing the timing of early COVID-19 infection compared to a community-based cohort, particularly during the first wave of the pandemic where community testing was not established. Thirdly, data for hip fracture patients in England are collected mandatorily by the NHFD as part of The Falls and Fragility Fracture Audit Programme (FFFAP), commissioned by the Healthcare Quality Improvement Partnership (HQIP) and managed by the Royal College of Physicians (RCP). As such, a wealth of individual level data is available (allowing for risk adjustment) for this cohort through linkage of NHFD data to established national data sources which are utilised in this study. Whilst we fully acknowledge that the mortality displacements seen in this population are not generalisable to the full English population, the hip fracture population is used as an ‘exemplar’ to demonstrate the end-to-end application of the methods presented.

## Methods

### Overview

We describe a novel method for estimating mortality displacement and how this is applied to adjust existing estimates of expected, and therefore excess mortality. We do not describe methods for modelling excess mortality in this paper. Instead, we describe how to use estimates of mortality displacement to adjust any existing estimate of excess mortality.

Methods for estimating mortality displacement are described in three steps.

1. Firstly, we estimate the combined hazard (risk of death) at every time-point for each individual in the modelled population who tested positive for COVID-19 (as it is only those individuals who developed COVID-19 that can have their death displaced by COVID-19) using a fitted Cox proportional hazards model. The Cox model is fitted based on all individuals in the population (not just those who tested positive for COVID-19) to allow the contribution of sociodemographic and health characteristics to mortality to be more precisely estimated. We use the hip fracture population as an exemplar population in our method rather than the whole English population. The model based on the hip fracture data describes the risk of death over time since the date of occurrence of hip fracture for each individual; the resulting hazards described by the model are, therefore, conditional upon suffering a hip fracture and are not generalisable to the full English population but allow the methodology to be demonstrated.
2. Secondly, for each individual in the modelled population who tested positive for COVID-19, we use the derived hazards to simulate a distribution of survival times, first in the presence of a COVID-19 positive test and, second, under the assumption that the same individuals had not tested positive. We use the difference between the median survival times for these two simulated scenarios to estimate the mortality displacement – i.e. number of days a death has been displaced.
3. In the final step, the mortality displacement estimates are then aggregated within sociodemographic and/or health characteristic groups that correspond to those used in existing population estimates of expected mortality in the population (e.g. by age, sex, and ethnicity groups). The aggregated distributions of mortality displacement estimates are then used to adjust estimates of excess mortality in the general population using a discrete probability model (which we refer to as a "ball and urn” model). The above steps are described in detail below.

#### Estimating the combined hazard

##### Cox proportional hazards model

Cox proportional hazards models (“Cox models”) are first used to explore the impact of demographic and health-related determinants on the risk of all-cause mortality in individuals in the defined study population [21]. The data structure must include the date of first COVID-19 positivity and the date of death, enabling models to disentangle and quantify the profile over time of the factors that increased risk of death following a positive COVID-19 test.

Cox models partition the risk of death at any given timepoint in the follow-up of a defined population of individuals into two components: (1) the fluctuating baseline hazard that, at any specified time, has a single value applying to everybody in the population; (2) the relative risk of death which is specific to each individual and is determined by the constellation of personal risk factors that that individual exhibits. The term hazard of death refers to a measure of the risk of death at a single point in time. The baseline hazard is analogous to the intercept/constant in a conventional regression model, but instead of a single value it exhibits different values at each distinctive follow-up time. Mathematically, it estimates the hazard of death at a given time-point for a hypothetical individual in whom all Cox model covariates take the value zero. In order to obtain the overall risk of death for a particular individual at that same time-point, the baseline hazard is multiplied by the product of the estimated multiplicative risks associated with all covariates that take values other than zero at that time-point in that individual.

Cox model covariates fall into two classes. Most are time-fixed: they have the same multiplicative effect on the hazard throughout follow-up. For example, as sex status remains unchanged throughout follow-up, a sex covariate is usually time-fixed. The other class is time-dependent. Here, the value of the covariate may change over time, or the covariate may remain fixed, but its estimated relative risk is allowed to change. The simplest covariate whose value may vary over time is the binary step function. At the start of follow-up this typically takes the value zero until a specified event occurs when it switches to one; it may return to zero at a later time. Binary step functions are central to our analysis. The time-dependent COVID-19 covariates all take a value zero when follow-up starts. As soon as any individual has a positive COVID-19 test their first COVID-19 covariate switches to value one. Two weeks later the first covariate switches back to zero and the second switches to one, while at four weeks the second switches back to zero and the third becomes one ….. etc. Having adjusted for all other modelled covariates, the coefficient associated with the first COVID-19 covariate reflects the multiplicative increase of death risk in the first two weeks after a positive COVID-19 test. Similarly, the second estimates the increased risk during weeks two to four etc*.* To be strictly formal the coefficients actually estimate the natural logarithm of the relative risks and require exponentiation to generate multiplicative effects.

By fitting a Cox model to the defined study population, we can obtain all analytic components needed to comprehensively interpret the survival profile of individuals and to tease out the temporal pattern of increased risk associated with COVID-19 infection. These key components are all captured in Eq. 1:


1

A failure time is a time-point in the follow-up where at least one individual dies. Equation 1 denotes the overall death hazard in subject $i$ at time $t$ as ${\lambda \left(t\right)}_{i}$. It is obtained by multiplying the baseline hazard at time $t$, *i.e.*  (  ), by the impact of all time-fixed covariates,  (  ), and again by the impact of all time dependent covariates,  (  ). In the term ${e}^{\left({{x}_{Fi}}^{T}{\beta }_{F}\right)}$, ${x}_{Fi}$ refers to a vector of values for all the time-fixed covariates in subject $i$, ${\beta }_{F}$ refers to the corresponding vector of time-fixed Cox regression coefficients. In this formula the superscript $T$ corresponds to the mathematical operation “multiply each covariate value by its corresponding coefficient and sum all the results”. Finally, exponentiation as denoted by $e$, renders the impact of each coefficient multiplicative. The equivalent term for the time dependent covariates  has the same interpretation except that the notation ${x}_{Di}(t)$ indicates that the value of each time dependent covariate must be evaluated at time $t$.

**Fig. 1:**
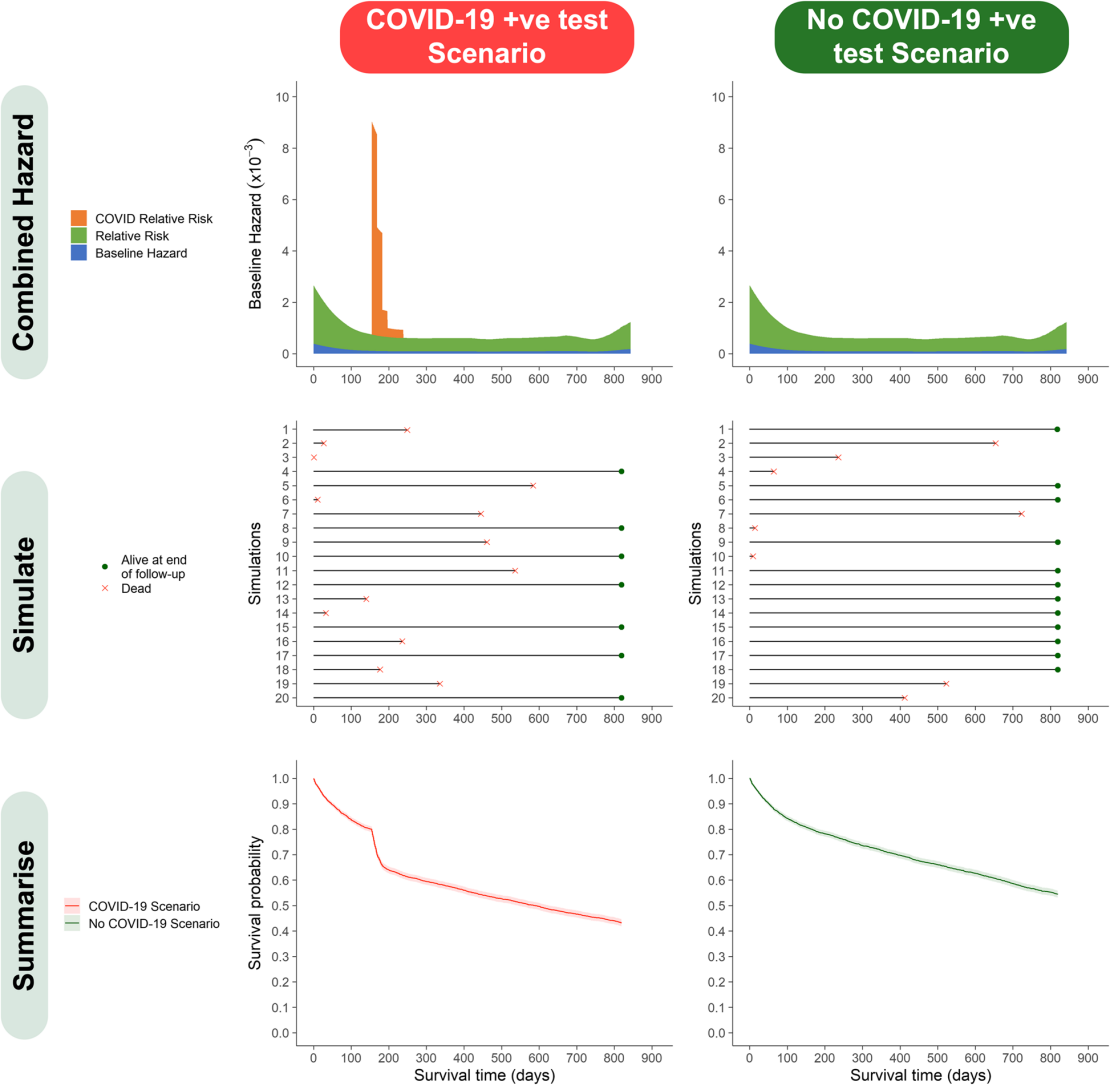
Simulation methodology—plot matrix describing simulation methodology for a hypothetical patient who developed COVID-19 infection 5 months following hip fracture. ‘Combined Hazard – illustrates the combined hazard for death for the example patient’s set of characteristics under COVID-19 positive test and no positive test scenarios. ‘Simulate’ – illustrates the results of the first 20 simulations of a patient’s survival times under positive test and no positive test scenarios. ‘Summarise’ – all individual simulation results for the example patient are summarised as a Kaplan–Meier cumulative survival function. Hazard refers to hazard of death. Relative Risk refers to the impact of the fixed and time-dependent covariate pattern for any individual. Source: Office for Health Improvement and Disparities, using data from National Hip Fracture Database (NHFD), Hospital Episode Statistics (HES) and Office for National Statistics (ONS), England. Copyright © 2022, Re-used with the permission of NHS Digital. All rights reserved

Cox models were all fitted using the coxph() function in R (“R: A Language for Data Analysis and Graphics”, version 4, Vienna, 2022). The basehaz() function in R is generally used to estimate the baseline hazard but cannot be applied to models with time-dependent covariates. We therefore used the estimator proposed by Breslow [22], and used our own R code to derive the baseline hazard at failure time $t$ as the number of failures at time $t$ divided by the sum of [  ] across all subjects alive at time $t$. Further details of the way in which our Cox models were fitted and used can be found in Supplementary Material A. The combined hazard for a hypothetical patient in our exemplar population who developed COVID-19 after a hip fracture is shown in Fig. 1.

### Exemplar study population

The Cox model should ideally be fitted on a cohort including all individuals within the population in which excess mortality is to be studied. We illustrate the application of this methodology using an exemplar population of patients who sustained a hip fracture. The exemplar study population included 98,365 patients aged > 60 years with a single hip fracture between 1/2/2019 and 31/10/20 included in England’s National Hip Fracture Database [18]. This represented 92% of all eligible patients with hip fractures in England over that period recorded national Hospital Episode Statistics (HES) [23]. These fracture data were linked pseudonymously at the level of individuals to HES (for patient characteristics and comorbidities), Office of National Statistics (ONS) mortality data (to determine date of death) [24], and to English SARS-CoV-2 antigen testing data (date of first positive COVID-19 test) to determine the time of testing positive for COVID-19 relative to the date of hip fracture (COVID-19 infection may occur before or after hip fracture) [25]. Individuals who sustained more than one hip fracture during the study period were excluded. Full details of data preparation, record linkage, and definitions of co-morbidities and frailty are described in a previous publication [20]. Date of death was used to derive survival time in days from the date of hip fracture presentation. Available patient characteristics and co-morbidities were modelled as time fixed terms in a Cox proportional hazard model; a time-dependent step function was used to define timing of an individual’s COVID-19 infection (if it occurred) relative to each risk-set time. Seasonality was adjusted for by defining each risk-set time according to season (spring, summer, autumn, winter) and year.

### Ethics approval and consent to participate

Study governance approval was granted by the FFFAP and UK Healthcare Quality Improvement Partnership (HQIP) in June 2020 (reference: HQIP286). National excess death modelling was carried out as part of Public Health England’s (PHE, now the Office for Health Improvement and Disparities (OHID)) responsibility to manage the COVID-19 pandemic. PHE/OHID have a legal basis, provided by Regulation 3 of The Health Service (Control of Patient Information) Regulations 2002, to process confidential patient information in order to monitor the impact of SARS-CoV-2 infection on the population and to respond to the pandemic.

#### Simulating from the Cox model

Equation 1 allows one to obtain ${\lambda \left(t\right)}_{i}$, the overall hazard of death for individual $i$ at failure time $t$. This permits a key inferential question central to a full counterfactual analysis. Specifically: by how much is the expected survival time reduced for an individual who contracts COVID-19 (actual life-experience) compared with what would have been expected had they not contracted COVID-19 (counterfactual life-experience)? The solution to this query is obtained by sequentially asking and answering a more fundamental mathematical question over a succession of time-points $t$: given that subject 𝒾 is still alive immediately before failure time $\mathrm{t}$, and regardless of whether they actually died or survived at time $\mathrm{t}$, what is the probability that individual 𝒾 would die at $\mathrm{t}$ rather than survive through $t$*?* Mathematically this is a frequentist probability (i.e. the long-run probability, if one could keep re-running reality) [26]; when the number of simulations of failure times for each individual is large, the asymptotic properties of treating each simulation as an independent replication result in probabilistic convergence of Eq. 1 to $1-{e}^{({-\lambda \left(t\right)}_{i})}$ [21, 22, 27]. The use of this result in our methodology is further described in Supplementary Materials B. A worked example of the derivation of the combined hazard from the Cox model for an example individual is shown in Table 1 and supported by Fig. 1.

**Table 1:**
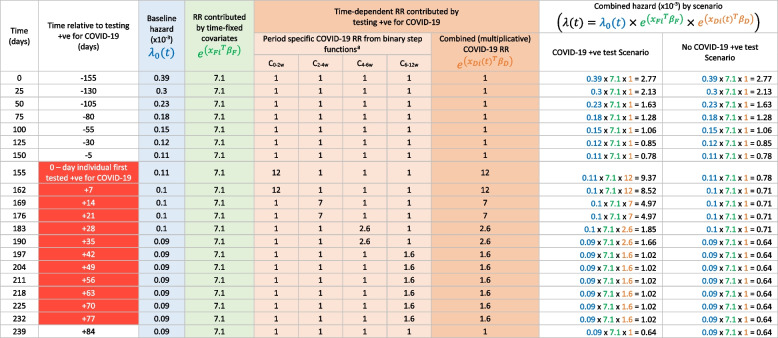
Worked example of the derivation of the combined hazard of death at example time-points for an individual who sustained a hip fracture and first tested positive for COVID-19 155 days after fracture

Time points are chosen to highlight the changes in COVID-19 time-dependent risk which occur after testing positive for COVID-19, according to binary step functions defined by a Cox proportional hazards model. The table illustrates how, at each time point after hip fracture, the combined hazard is calculated by multiplying the baseline hazard by the relative risk for time-fixed covariates (chosen in this example to include a range of co-morbidities conferring a relative risk of 7.1) and multiplying again by the relative risk for the time-varying exposure of testing positive for COVID-19

*RR* Relative risk

^a^ The relative risk for testing + ve for COVID-19 is described by four binary step functions which ‘turn on’ as the individual enters four pre-defined time-windows of varying COVID-19 risk – namely from 0 to 2 weeks (C_0-2w_, RR = 12.1), 2 to 4 weeks (C_-2-4w_, RR = 7), 4 to 6 weeks (C_4-6w_, RR = 2.6), and 6 to 12 weeks (C_6-12w_, RR = 1.6) following testing + ve for COVID-19 and return to a relative risk of 1 once the individual moves outside the time window defined by the step function

Using the theory outlined, it is straightforward to repeatedly simulate the expected date of death of any given individual in the study population and thereby to obtain a full probability distribution for that date of death. We start with individual 𝒾 = 1 and focus on the first unique failure time observed in the study population ($${uft}_{1}$$). We generate a pseudorandom number ($$r$$) from a uniform distribution between 0 and 1. If $$r\le$$$${\lambda \left(1\right)}_{1}$$ individual 1 is viewed as having died at $${uft}_{1}$$ otherwise they pass on to $${uft}_{2}$$ and the simulation process is repeated, and so on. If subject 1 is ultimately simulated to have died at the failure time corresponding to time $$u$$, his/her date of death in simulation 1 is declared as $$u$$: *i.e. *$${date.of.death}_{[\mathrm{1,1}]}=u$$. If it has been decided, a-priori, to undertake M simulations the same approach can now be used to create a total of M independent realisations of the expected date of death for individual 1: *i.e. *$${date.of.death}_{[\mathrm{1,1}]}$$, ….., $${date.of.death}_{[1,M]}$$. Once the simulations for individual 1 are complete the same process is repeated across all N subjects in the study population thus obtaining a total of $$N\times M$$ simulated times of death where $${date.of.death}_{[i,s]}$$ is the simulated date of death in the $${s}^{th}$$ simulation for the $${i}^{th}$$ individual. If at any simulation an individual survives through every failure time, they are designated as censored (still alive) at the last failure time.

#### Estimation of displacement

Using the theory outlined above and the information contained in the output of the Cox model and Eq. 1, we repeatedly simulate and compare the expected survival profile (for the exemplar hip fracture population, this is the survival time from the date of hip fracture until the date of death) of all individuals in the modelled population who tested positive for COVID-19 under two distinct scenarios:

1. The ‘COVID-19 positive test scenario’ (i.e. what actually occurred*:* some members of the study population tested positive to COVID-19 infection). These simulations are based on the full Cox model.
2. The ‘no COVID-19 positive test scenario’ (i.e. the counterfactual scenario*:* no members of the study population tested positive to COVID-19 infection). These simulations are based on the same Cox model (all non-COVID-19 covariates remain the same) but with all COVID-19 covariates set to 0 for all subjects at all times.

Figure 1 – “simulate” shows the results of the first 20 simulations for a hypothetical patient, “summarise” shows a Kaplan–Meier survival curve derived using all M simulation results as pseudo-observations. For each individual, the M simulations reflecting the expected date of death under the two scenarios is summarised by their respective medians. The difference between these two medians, ${\Delta }_{i}$, is then a measure of the expected shift (generally death would have been brought forward to an earlier time by testing positive for COVID-19) in the date of death under the actual COVID-19 positive test scenario compared with the counterfactual *no positive test* scenario. The quantity ${\Delta }_{i}$ may be referred to as the mortality displacement in individual 𝒾. The median is chosen to characterise the survival distributions rather than the mean because survival distributions typically have long right-hand tails. The rationale for comparing simulated actual and counterfactual medians is presented in Supplementary Material C.

In order to estimate the median date of death under either scenario, the M simulated dates of death (and censoring statuses to incorporate those that survive through all failure times) in individual 𝒾 are treated as if they were M survival times across M individuals in a study population and summarised using a conventional Kaplan–Meier plot. If the Kaplan–Meier survival function in subject 𝒾 falls below 50%, the median is obtained as the particular survival time at which the survival function first dropped below 50%. On the other hand, if the survival function never falls to 50% no direct estimate exists for the median survival time. To address this challenge, the survival curve is first linearised by taking the natural logarithm of the survival probabilities. The last 25% of the curve is then extrapolated based on the best fitting straight line and the median survival is identified as the survival time at which that straight line falls below a log_e_(probability of survival) of log_e_(0.5) = -log_e_(2) ~ -0.693. Further details are provided in Supplementary Material D.

#### Applying the mortality displacements to adjust estimates of excess mortality

Finally, the distribution of the individually estimated mortality displacements (${\Delta }_{i}$) is used to adjust the number of deaths predicted to occur in any given week based on the conventional methods that had been used pre-COVID-19 to calculate expected mortality rates in the general population and thus derive the extent of excess mortality [6].

**Fig. 2:**
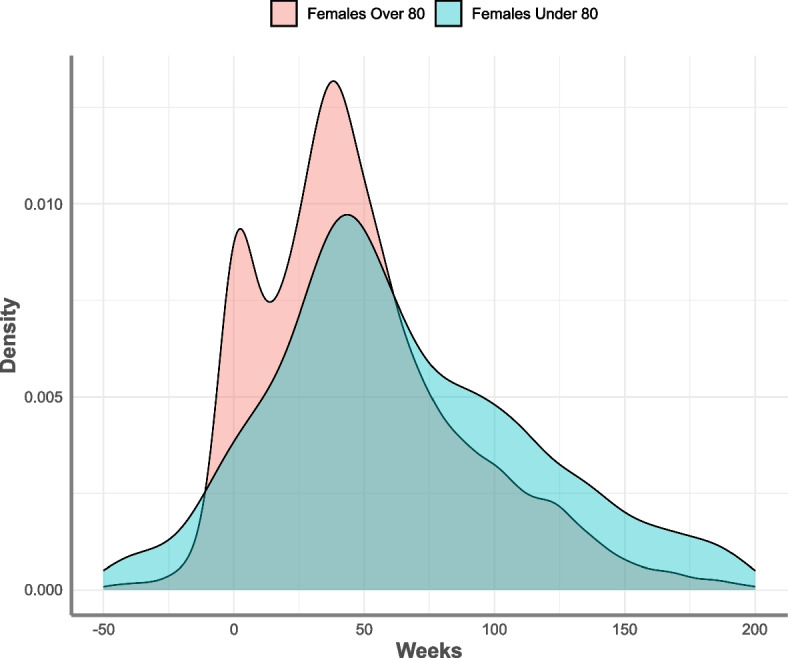
Density distribution of simulated median survival time differences between COVID-19 positive test and no-COVID-19 positive test scenarios, for hip fracture in female patients by age. Source: Office for Health Improvement and Disparities, using data from National Hip Fracture Database (NHFD), Hospital Episode Statistics (HES) and Office for National Statistics (ONS), England. Copyright © 2022, Re-used with the permission of NHS Digital. All rights reserved

**Fig. 3:**
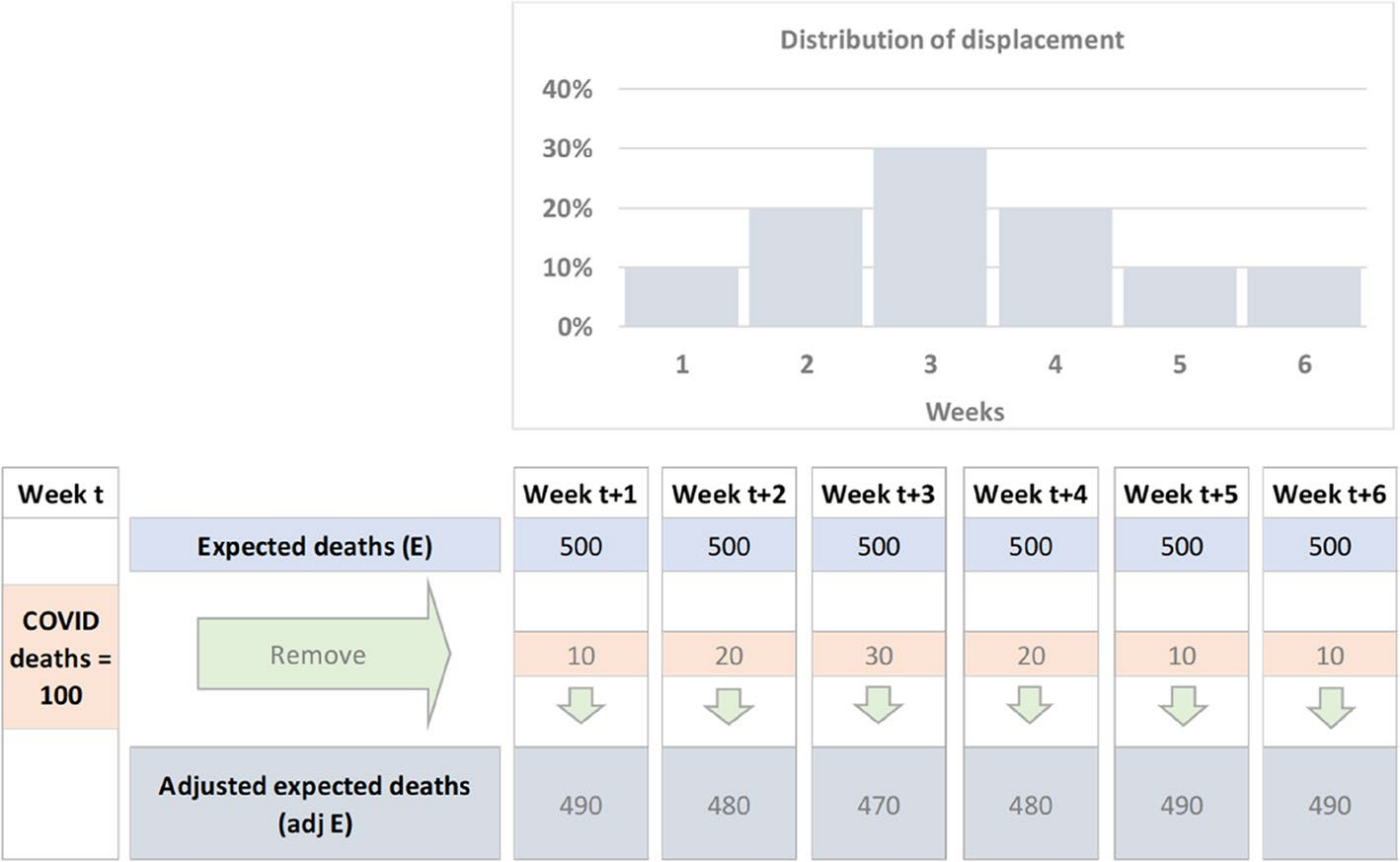
Simple example for adjusting expected deaths using displaced COVID-19 deaths

For this final step, three sets of metrics are required: 1. the empirical distribution of mortality displacement generated through the steps described in the sections above; 2. COVID-19 deaths at a given time $t$ (it is important to note that, at the time of writing, nationally reported weekly COVID-19 deaths by the Office for National statistics were defined by death certificate mentions and not COVID-19 testing); and 3. estimates of expected deaths at $t$ from PHE's (now OHID) original excess deaths model [3]. For this step we adopt a heuristic approach to adjusting the expected number of deaths in any given week, based on the difference in expected survival time under positive test and NO-positive test scenarios—${\Delta }_{i}$—applied to people who died from COVID-19 in week $t$. In the simplest case each of these deaths is randomly allocated a displacement time based on the empirical distribution of the modelled survival time differences ${\Delta }_{i}$ (see Fig. 2). The subsequent analysis is perhaps best illustrated via a practical simplified example based on a “ball and urn” model as outlined in Fig. 3.

In the simplified example illustrated in Fig. 3, the empirical distribution of ${\Delta }_{i}$ is constructed to fall between one and six weeks, with no negative values of ${\Delta }_{i}$ being observed. In fact, in this simplistic example, 10% of the deaths are positively displaced by one week, 20% by two weeks, 30% by three weeks, 20% by four weeks, 10% by 5 weeks and 10% by six weeks. Using this empirical distribution, the expected deaths in weeks $t$+1,…, $t$+6 are adjusted by subtracting the corresponding number of COVID-19 deaths which occurred during week $t$ which would have expected to be displaced to this time, resulting in an adjusted expected deaths estimate. This process is carried out iteratively, week by week, on each occasion removing the appropriate number of expected deaths from the down-stream weeks where they would have been expected to fall in the absence of COVID-19 deaths.

For the current analysis we apply displacement by creating the discrete probability distribution of the modelled survival time differences by week ${\Delta }_{i}$ for 4 strata within the hip fracture patients. The strata were: greater than or equal to 80 years vs under 80 years; by male and females. Each distribution represents the time adjustment for COVID-19 deaths within that stratum for any week in time where a death was reported. For each week $t$ when deaths where a cause of COVID-19 occurred, the total deaths were re-distributed in time according to the probabilities for each week of the strata survival distribution ${\Delta }_{i}$, before being removed from the corresponding strata expected deaths for weeks $t$+1, …, $t$+6. COVID-19 reported deaths start at week 0 (27^th^ March 2020) and for this analysis we have used a final cut-off week of the 8^th^ of October 2021 for reported COVID-19 deaths.

Following adjustment of expected deaths in each week by stratum, the estimate of excess mortality in each week is re-estimated as the observed number of deaths in that week minus the adjusted expected number of deaths (see Supplementary Material E for further detail). The key steps of our method are summarised in Table 2 using mathematical notation.

**Table 2:**
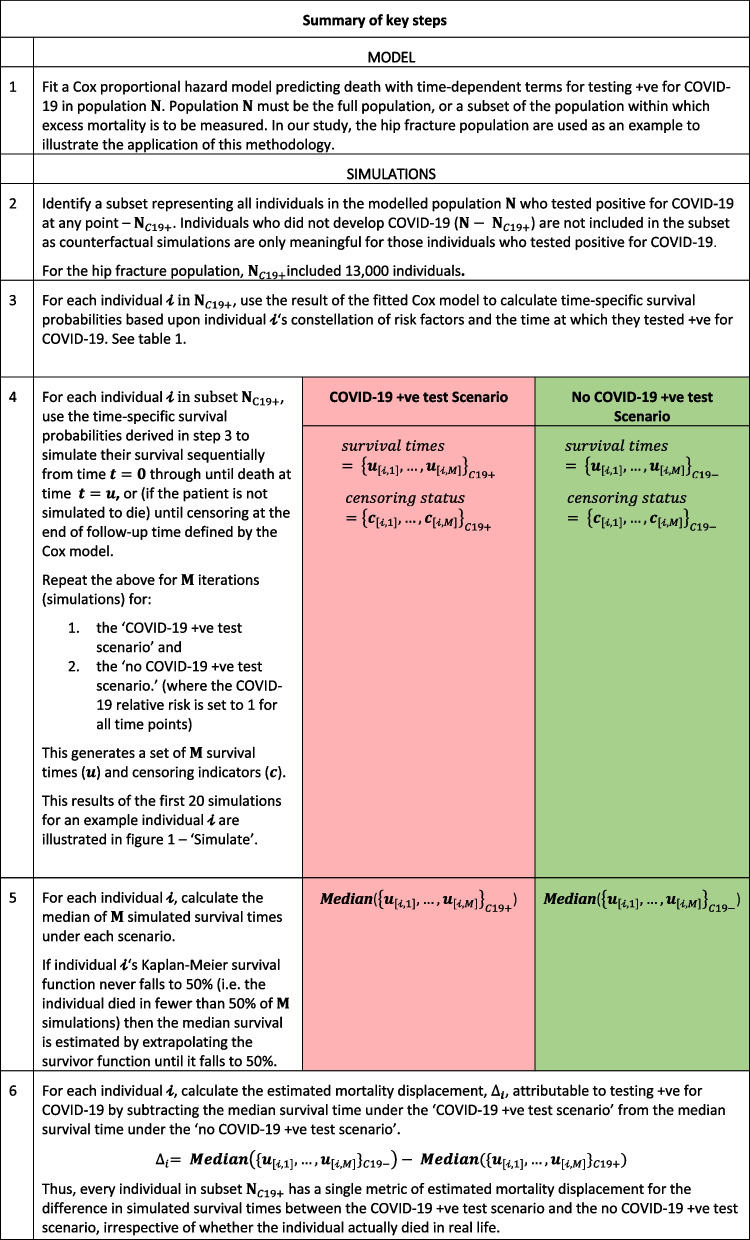

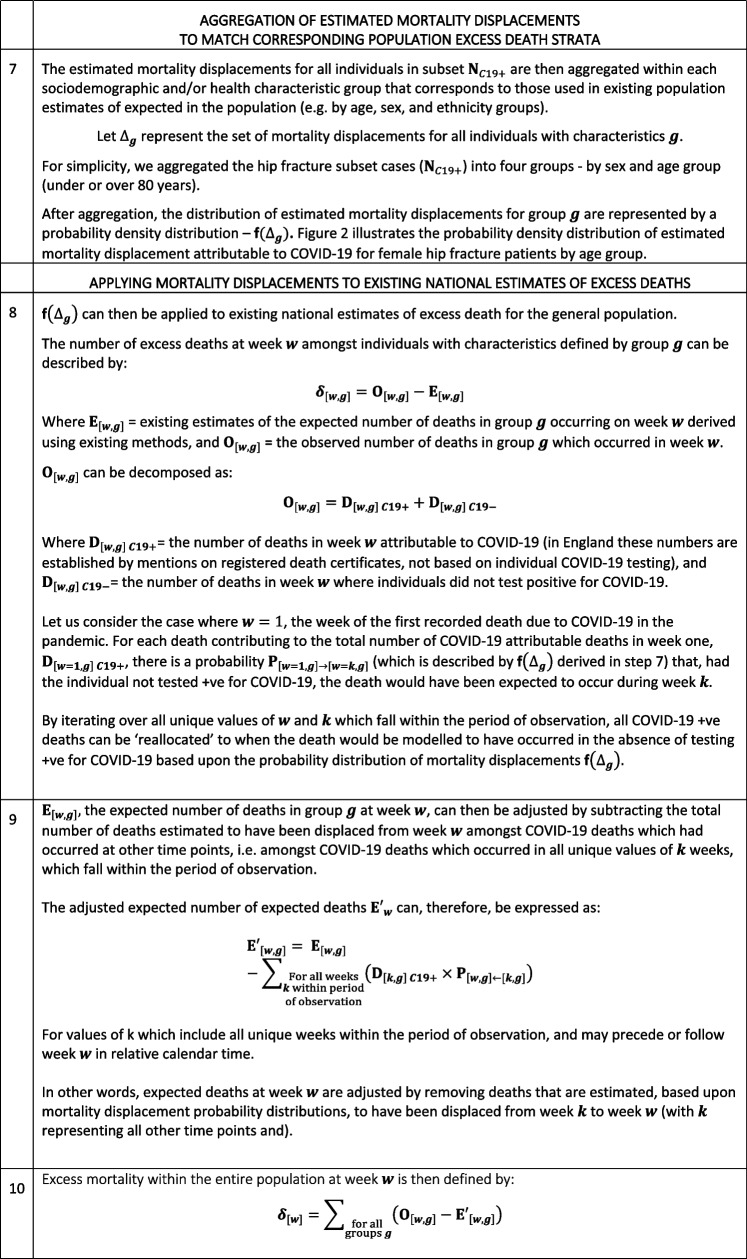
Summary of the end-to-end application of the methods described in this study to adjust national estimates of expected deaths

## Results

**Fig. 4:**
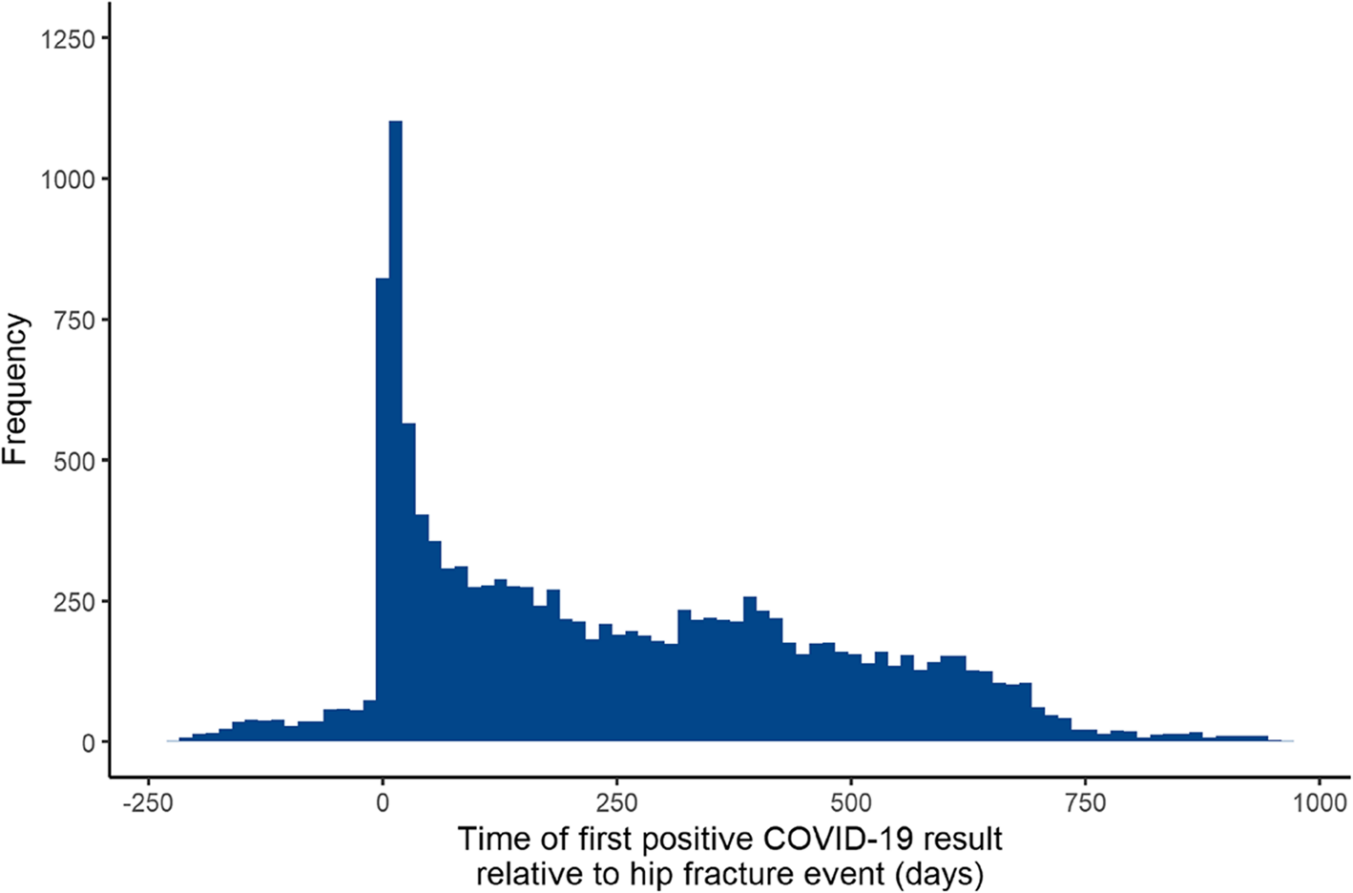
Distribution of timing COVID-19 infection (first recorded positive test) relative to hip fracture (grouped into 14-day period bin-widths). Source: Office for Health Improvement and Disparities, using data from National Hip Fracture Database (NHFD), Hospital Episode Statistics (HES) and Office for National Statistics (ONS), England. Copyright © 2022, Re-used with the permission of NHS Digital. All rights reserved

The study population included 98,365 hip fracture patients, in the period 1^st^ February 2019 to 31^st^ October 2020, with 13,000 of the affected patients developing COVID-19 before or after hip fracture. The distribution of timing of COVID-19 positive tests relative to hip fracture (time-dependent term) is shown in Fig. 4. Most of those individuals who tested positive for COVID-19 did so following hip fracture with a peak at, or shortly after the date of their admission; this is partly a detection bias. Patient and surgical characteristics for the cohort are shown in Table 3 broken down by COVID-19 and mortality status.

**Table 3:** Characteristics of study population by COVID-19 and followed up for mortality status until 31^st^ May 2021

		**COVID-19 Positive** **n (%)**		**COVID-19 Negative** **n (%)**		**Total** **n (%)**
**Variable**	**Level**	**Alive**	**Dead**	**Alive**	**Dead**	
**Total**		7,711 (7.8)	5,289 (5.4)	56,211 (57.1)	29,154 (29.6)	98,365
**Time from hip fracture to COVID-19 infection (days)**	**Mean (SD)**	279.3 (238.3)	204.7 (204.7)	n/a	n/a	249.0 (228.2)
**Age group (years)**	**60 to 69**	514 (6.7)	149 (2.8)	6,403 (11.4)	1,155 (4.0)	8,221 (8.4)
	**70 to 79**	1,667 (21.6)	876 (16.6)	16,311 (29.0)	4,498 (15.4)	23,352 (23.7)
	**80 to 89**	3,742 (48.5)	2,568 (48.6)	24,547 (43.7)	13,384 (45.9)	44,241 (45.0)
	**90 + **	1,788 (23.2)	1,696 (32.1)	8,950 (15.9)	10,117 (34.7)	22,551 (22.9)
**Sex**	**Female**	5,787 (75.0)	3,144 (59.4)	41,698 (74.2)	18,156 (62.3)	68,785 (69.9)
	**Male**	1,924 (25.0)	2,145 (40.6)	14,513 (25.8)	10,998 (37.7)	29,580 (30.1)
**Residence**^a^	**Own home/sheltered housing**	5,305 (68.8)	3,358 (63.5)	50,401 (89.7)	19,019 (65.2)	78,083 (79.4)
	**Residential care**	1,327 (17.2)	970 (18.3)	3,007 (5.3)	4,985 (17.1)	10,289 (10.5)
	**Nursing care**	816 (10.6)	681 (12.9)	1,776 (3.2)	3,506 (12.0)	6,779 (6.9)
	**Hospital inpatient**	263 (3.4)	280 (5.3)	1,027 (1.8)	1,644 (5.6)	3,214 (3.3)
**Mobility**^a^	**Freely mobile without aids**	2,253 (29.2)	1,100 (20.8)	26,815 (47.7)	6,145 (21.1)	36,313 (36.9)
	**Mobile outdoors with one or more aids or a frame**	2,961 (38.4)	2,174 (41.1)	19,970 (35.5)	11,304 (38.8)	36,409 (37.0)
	**Never goes outside without help or no functional mobility**	2,397 (31.1)	1,939 (36.7)	9,089 (16.2)	11,252 (38.6)	24,677 (25.1)
	**Not recorded**	100 (1.3)	76 (1.4)	337 (0.6)	453 (1.6)	966 (1.0)
**Nutrition**^a^	**Normal nutrition**	5,488 (71.2)	3,567 (67.4)	44,028 (78.3)	18,223 (62.5)	71,306 (72.5)
	**At risk of malnutrition**	1,522 (19.7)	1,141 (21.6)	8,155 (14.5)	6,869 (23.6)	17,687 (18.0)
	**Malnourished**	498 (6.5)	429 (8.1)	2,854 (5.1)	3,132 (10.7)	6,913 (7.0)
	**Not recorded**	203 (2.6)	152 (2.9)	1,174 (2.1)	930 (3.2)	2,459 (2.5)
**Hospital Frailty Risk Score group**	**Low risk (< 5)**	496 (6.4)	169 (3.2)	9,569 (17.0)	1,252 (4.3)	11,486 (11.7)
	**Intermediate risk (5–15)**	2,888 (37.5)	1,686 (31.9)	29,213 (52.0)	9,498 (32.6)	43,285 (44.0)
	**High risk (> 15)**	4,327 (56.1)	3,434 (64.9)	17,429 (31.0)	18,404 (63.1)	43,594 (44.3)
**Cardiovascular disease**	**Yes**	3,526 (45.7)	3,054 (57.7)	19,646 (35.0)	16,589 (56.9)	42,815 (43.5)
**Malignancy**	**Yes**	690 (8.9)	791 (15.0)	5,039 (9.0)	5,792 (19.9)	12,312 (12.5)
**Dementia**	**Yes**	3,236 (42.0)	2,458 (46.5)	9,903 (17.6)	13,283 (45.6)	28,880 (29.4)
**Chronic pulmonary disease**	**Yes**	2,287 (29.7)	1,714 (32.4)	14,932 (26.6)	10,024 (34.4)	28,957 (29.4)
**Liver disease**	**Yes**	271 (3.5)	218 (4.1)	1,794 (3.2)	1,293 (4.4)	3,576 (3.6)
**Diabetes (Type 1 & 2)**	**Yes**	1,642 (21.3)	1,298 (24.5)	10,222 (18.2)	6,377 (21.9)	19,539 (19.9)
**Hemiplegia or paraplegia**	**Yes**	265 (3.4)	185 (3.5)	1,562 (2.8)	944 (3.2)	2,956 (3.0)
**Renal disease**	**Yes**	2,003 (26.0)	1,861 (35.2)	10,930 (19.4)	9,948 (34.1)	24,742 (25.2)

^a^ At time of presentation with hip fracture. Source: OHID, using data from National Hip Fracture Database (NHFD), Hospital Episode Statistics (HES) and Office for National Statistics (ONS), England. Copyright © 2022, Re-used with the permission of NHS Digital. All rights reserved

### Multivariate model

**Fig. 5:**
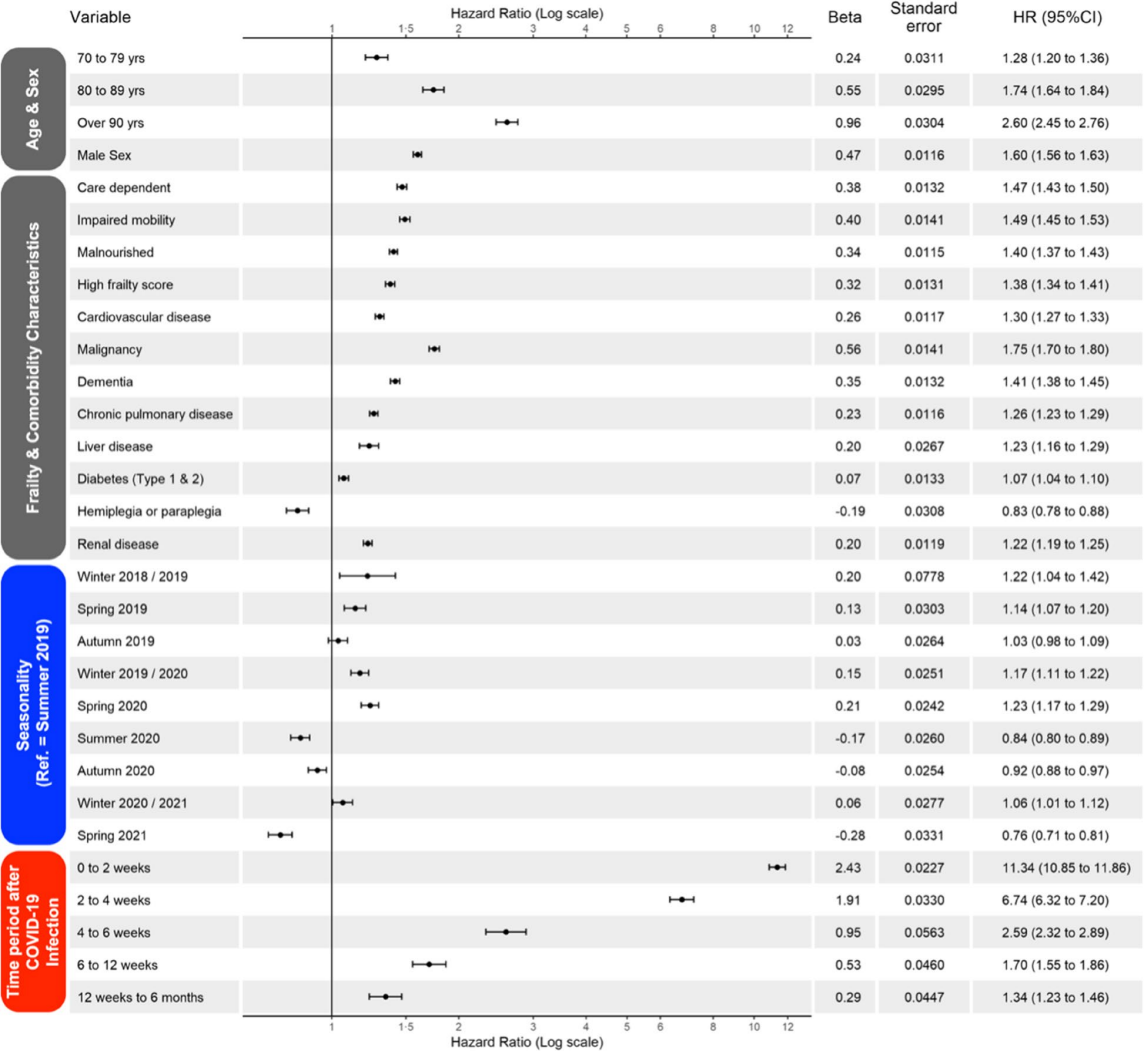
Coefficient plot for multivariate Cox proportional hazard model with time fixed and time dependent terms. Reference groups: Age = 60–69 years; Frailty = low or intermediate frailty, Co-morbidity = absence of the condition; Seasonality = summer 2019. Seasonality defined as Winter (months December, January, February), Spring (March, April, May), Summer (June, July, August), Autumn (September, October, November). Source: Office for Health Improvement and Disparities, using data from National Hip Fracture Database (NHFD), Hospital Episode Statistics (HES) and Office for National Statistics (ONS), England. Copyright © 2022, Re-used with the permission of NHS Digital. All rights reserved

Results of the multivariate Cox proportional hazard model are shown in Fig. 5, including time-fixed terms and COVID-19 time-dependent terms. The hazard of death was greatest within the first two weeks of testing positive for COVID-19 and gradually declined until almost reaching baseline risk by six months.

### Estimation of mortality displacement

**Fig. 6:**
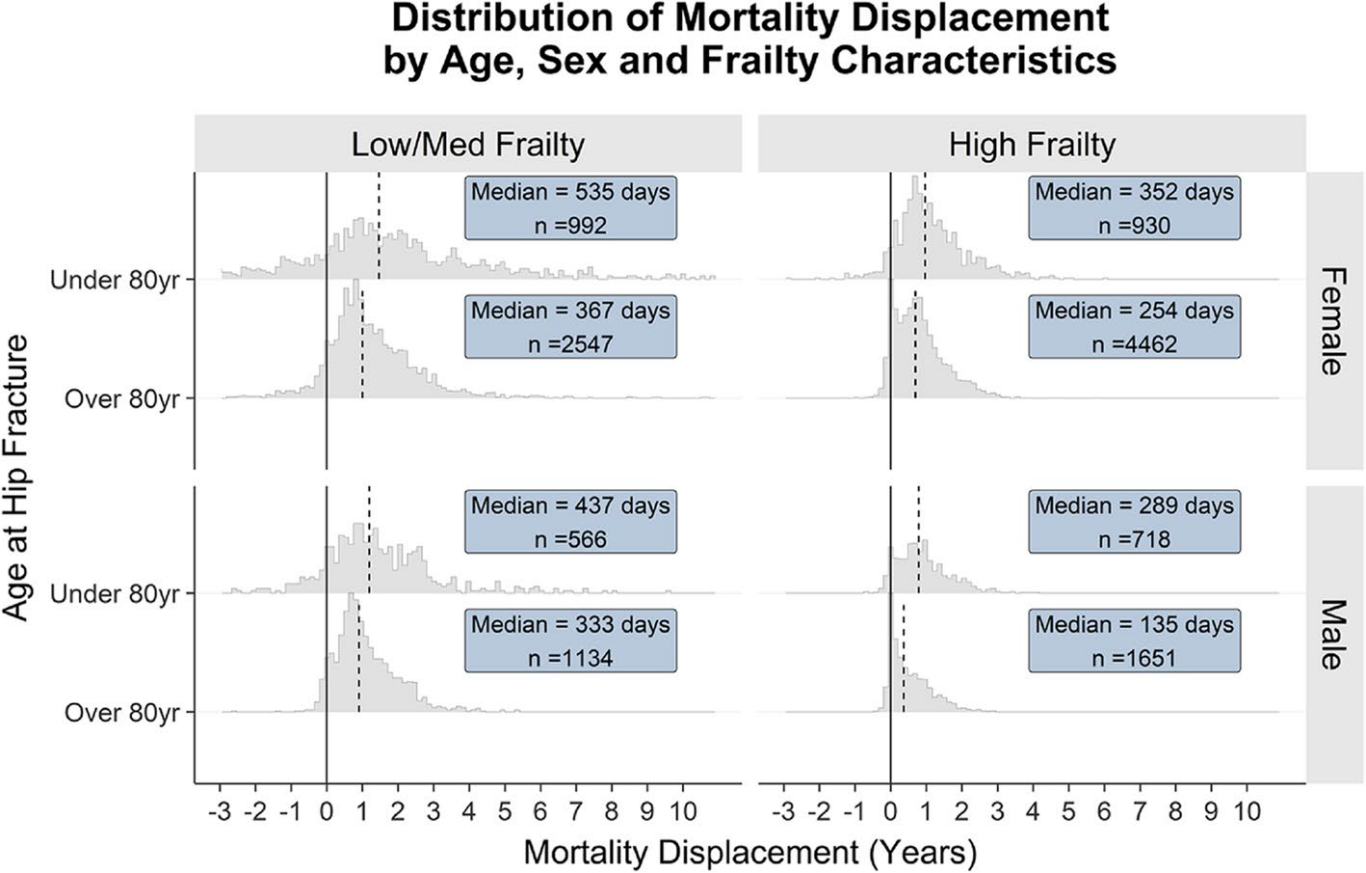
Histogram of mortality displacements in the hip fracture cohort stratified by age, sex, and frailty characteristics (high frailty = hospital frailty risk score ≥ 15). Dashed vertical line illustrates median displacement. Text box shows median displacement and number of individuals within each stratum. Source: Office for Health Improvement and Disparities, using data from National Hip Fracture Database (NHFD), Hospital Episode Statistics (HES) and Office for National Statistics (ONS), England. Copyright © 2022, Re-used with the permission of NHS Digital. All rights reserved

Five hundred simulated actual and counterfactual survival times for each hip fracture who tested positive were summarised using the median survival time (extrapolated where necessary for those whose survival estimate did not reach 50% at the time of maximum simulated follow-up). Mortality displacements due to COVID-19 positive infection were calculated for each hip fracture as the difference between these two summarised values and presented as distributions grouped by age, sex, and frailty characteristics (Fig. 6). Mortality displacement was smallest for older, frail, and male individuals (median = 135 days) and was largest for younger, female and lower frailty individuals in deaths were on average brought forward due to COVID-19 by almost 2 years (median = 535 days).

### Adjusting expected and excess mortality estimates

**Fig. 7:**
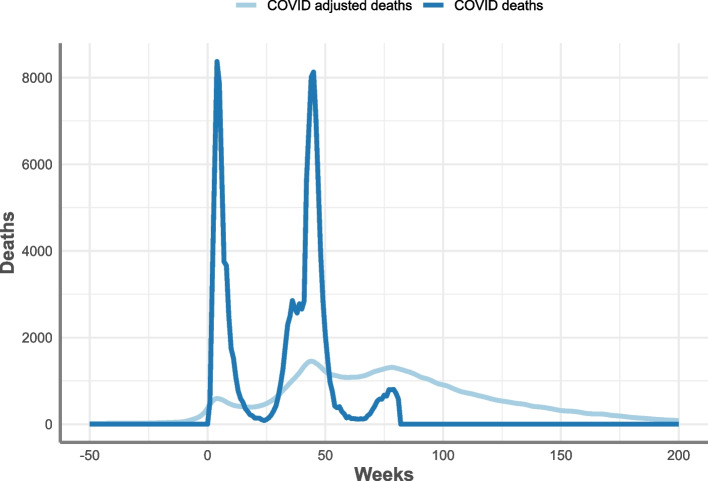
Total COVID-19 registered deaths and adjusted COVID-19 deaths from week 9 (27^th^ March 2020) to week 81 (8^th^ October 2021), England. Source: Office for Health Improvement and Disparities, using data from National Hip Fracture Database (NHFD), Hospital Episode Statistics (HES) and Office for National Statistics (ONS), England. Copyright © 2022, Re-used with the permission of NHS Digital. All rights reserved

The median difference between the actual and counterfactual survival times for each of the 13,000 COVID-19 patients with hip fractures were used to create a discrete probability distribution for four strata. Registered deaths in England with a cause of COVID-19 between 27^th^ March 2020 and 8^th^ October 2021 were adjusted in time using the probability distributions, with 48.4% of total COVID-19 deaths remaining within the time period after adjustment (Table 4 and Fig. 7). Removing adjusted COVID-19 deaths from the published expected number reduced expected deaths by 8.9% over this time period (Table 4).

**Table 4:** Total registered COVID-19 deaths, modelled expected deaths and excess mortality between 27^th^ March until the 8^th^ October 2021, including adjusted figures using hip fracture COVID-19 mortality displacement, England

	**Total**	**Adjusted**
**Expected deaths**	763,448	695,425
**Excess mortality**	102,663	170,686

Source: Office for Health Improvement and Disparities, using data from National Hip Fracture Database (NHFD), Hospital Episode Statistics (HES) and Office for National Statistics (ONS), England. Copyright © 2022, Re-used with the permission of NHS Digital. All rights reserved

**Fig. 8:**
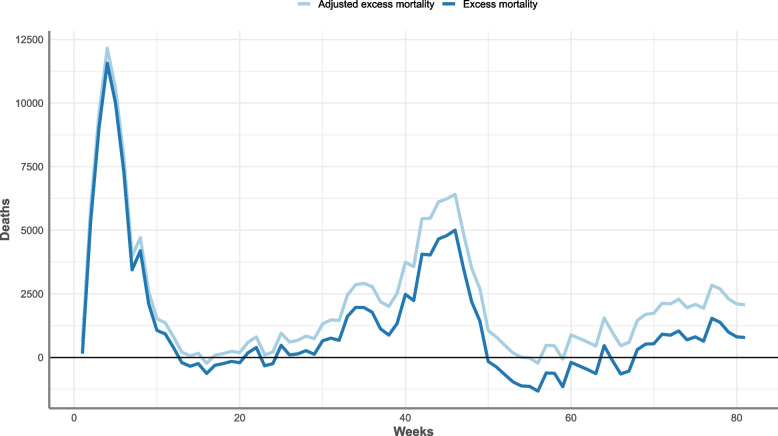
Excess mortality and adjusted excess mortality from week 1 (27^th^ March 2020) to week 81 (8^th^ October 2021), England. Source: Office for Health Improvement and Disparities, using data from National Hip Fracture Database (NHFD), Hospital Episode Statistics (HES) and Office for National Statistics (ONS), England. Copyright © 2022, Re-used with the permission of NHS Digital. All rights reserved

An example of adjusting the published excess mortality for England using the adjusted expected deaths (based on the hip fracture data) is shown in Fig. 8 from the period of 27^th^ March 2020 until the 8^th^ October 2021. Over this time period the total excess deaths increased by 66% (Table 4), with the difference between excess mortality and adjusted excess mortality increasing each week over this period. The number of weeks in which the excess mortality was estimated to be negative reduced from 27 to 5 weeks after adjustment (Fig. 8).

## Discussion

Using a national dataset of hip fracture patients as an illustrative dataset, we describe and demonstrate a novel and generalizable method for estimating the parameters underpinning mortality displacement consequent upon COVID-19 positive test. We then use these parameters to adjust expected mortality, and thereby excess mortality (the difference between the number observed and the number expected in any time period), to facilitate on-going surveillance of excess mortality during the remaining pandemic and beyond.

Our results, based on the hip fracture dataset, indicate that estimating the current expected number of deaths based solely on historical trends is an overestimate because many of the deaths expected on this basis would already have occurred earlier in the pandemic. This means that, over any period well after the start of the pandemic, calculating the excess deaths based on historical trends will be an underestimate. These both represent direct consequences of what may be called mortality displacement or harvesting.

Although our results provide clear evidence of short-term mortality displacement in the population included in the national hip fracture registry, interpretation of its magnitude needs to take account of the fact that this is a select subgroup of the general population. Fractures that impede mobility in the weeks that follow the accident/surgery considerably shorten life expectancy – at least in the elderly – and are in themselves markers of significant frailty [13, 28]. In our analysis we have demonstrated that age, sex, and frailty all have substantial impacts on the frequency and magnitude of mortality displacement. This implies, given that our ultimate aim is to generate quantitative estimates of the effect of mortality displacement in the general population, that it is important that we apply these methods to a representative sample of the national population. The critical importance of the analyses reported in the current methodological paper is to demonstrate the methodology, working through it from end-to-end, and giving us the confidence to engage in the major task of applying them to national population data.

In interpreting the results of our analyses, it is individuals with low frailties and the largest displacements that have almost no impact on short term estimates of expected deaths (*e.g.* deaths over the next 12 months). This is because, with reference to Fig. 3, an individual that died of COVID-19 in a given week $t$, who would have been expected to live for a further 520 weeks (10 years) under the counterfactual, would only impact on the count of expected deaths at $t$+520 weeks. In contrast, the subpopulations in which mortality displacement has the greatest effect on current, past, and near-future estimates of expected mortality are older people with large frailties in whom statistical power is high, because there are so many deaths. Therefore, among this group, the magnitude of displacement can be estimated more precisely. Although the magnitude of displacement in younger, low-frailty subpopulations can only be estimated with low precision, the precise magnitude is less important when adjusting current expected mortality estimates, because there is considerable certainty that individuals in this group would have survived well beyond the end of the prediction interval.

These observations have important implications for the use of our method in practice. First, it is adequately powered for the short-term adjustment of expected mortality. Specifically, the estimates that are generated are more precise in frailer elderly subgroups because there are more deaths, but the lack of precision in younger fitter populations will appropriately be reflected in wide confidence intervals which should guard against over-interpretation. Second, estimates of the hazard ratios (relative risks) associated with COVID-19 infection and their profile over time after infection are estimated with precision. This means these hazard ratios are useful as epidemiological metrics that can feed directly into our understanding of the public health implications of COVID-19. Third, however, it is less useful for what might be another apparently attractive use of our method—to estimate years of life lost (YLL). In the $i$^th^ individual this is obtained directly as ${\Delta }_{i}$ (see above). Because this is so straightforward and given that YLL is *“*a frequently used population health metric, originating back to the 1940s … and the idea is appealingly simple*”*,^*7*^ it sounds like an ideal use of method. But YLL requires very careful interpretation. It is true that by applying our method across all age groups and summing the ${\Delta }_{i}$ one could generate an overall point estimate of YLL, and by restricting this to people who died of COVID-19 and dividing the total by the number of individuals one could obtain a point estimate for average YLL per COVID-19 death to compare to other equivalent estimates [29–32]. However, this would be of limited value. In subpopulations that are old or frail we can generate estimates of YLL with acceptable precision, and this could provide a useful public health metric of the impact of the pandemic in those frailer subpopulations. But in younger fitter subpopulations although we can confidently state that the relatively small number of individuals who died of COVID-19 lost many years of life, any formal quantification of those YLL would be too imprecise to be of value. Rather, in younger populations it is more sensible to estimate YLL using the “WHO Standard Approach” [30], based on applying an appropriate standard life table to people who died of COVID-19 [29]. We therefore believe that our methods, particularly once they have been applied to a nationally representative population, can provide a useful contribution to a description of YLL in older and frailer subpopulations. But we would not recommend they be used, on their own, to attempt to generate an estimate for the average YLL per COVID-19 death across the entire population which is better obtained in other ways [29–32].

The application of Cox hazards models with time-varying covariates to simulate survival times has been validated previously [33]. Model-based approaches – such as ours – have been used previously to explore short and long-term displacement of mortality caused by exogenous events such as extreme temperatures, air pollution and influenza seasons. These have generally adopted deterministic and stochastic lag models [16, 17, 34], based on treating mortality at a population level as a Poisson (or Quasi-Poisson) outcome [35]. Analogous population-level approaches have been used in publications looking at the extent of mortality displacement that had occurred leading up to the pandemic and the subsequent impact on pandemic mortality and indicate some level of displacement [8, 35–39]. In England and France short-term mortality displacement has been estimated at a population level by comparing the number of deaths above the expected value to those below the expected value within the given time period [8, 38]. In a USA study, no mortality displacement was identified, however, among other issues, as the study period ended in May 2020, this may be a result of the short follow-up [35].

One method which allows for the estimate of displacement at an individual level to estimate years of life lost [40], takes the distribution of excess mortality by age group and applies the number of years of life expectancy lost in each group [41]. This method has been applied to data in Sweden and Norway, with the authors making adjustments to expected mortality [41]. The study estimated YLL attributable to COVID-19 in 2020 being 45,850 without adjustment for pre-pandemic seasonal influenza mortality displacement and 43,073 when adjusted for displacement. In Scotland, the number of YLL was estimated to approximately 15 per COVID-19 death in 2020 [42]. In the USA, authors used projections of life expectancy under different COVID-19 scenarios to identify variation in outcomes between age and ethnic groups [43]. For example, in a medium COVID-19 scenario the estimated reduction of life expectancy at birth in 2020 was approximately 1.13 years with large ethnic disparities – life expectancy for Black and Latino groups was estimated to be reduced by 2.10 and 3.05 years respectively, compared with 0.68 years for White groups. However, these methodologies are limited to analysis among groups for whom life expectancy is calculated and therefore is unable to account for the complex interactions between co-morbidities and COVID-19 infection and risk of death. They, therefore, also assume that those who contract COVID-19 are a random subset of the population; yet evidence suggests a higher susceptibility of COVID-19 infection amongst more vulnerable subgroups [44, 45].

### Limitations

We present the model in this paper as proof of concept, though it can be extended to include as much detail on risk factors as is available within the data source. It does have some limitations – it ultimately depends on accurately identifying those testing positive for COVID-19 infection, which is problematic when analysing mortality for early pandemic periods when, for many countries there was limited testing. This may also become more problematic as the method is applied to later pandemic periods where national policy decisions on testing impact the ability to collect accurate testing numbers. The method does not address displacement among those dying with COVID-19 but without a positive test, mortality that occurred because of indirect effects of the COVID-19 pandemic either on the health system, such as limited access to services, or other effects of containment measures. In both cases these groups will have been included among the non-COVID-19 deaths, resulting in an underestimate of mortality displacement. It also does not address the problem of disruption to historic trends for other reasons, therefore, does not attempt to estimate what would have occurred in the absence of the pandemic. Several approaches can be applied in parallel to address many of these questions. Theoretically, the approach depends on knowing all key variables, which is, as in all modelling situations, impossible to obtain. Additionally, estimation of the extent that deaths are deferred is limited to what is observed within the follow-up period. Our “ball and urn” method for adjusting expected deaths using counts of registered deaths where COVID-19 is mentioned on the death certificate (as opposed to formal COVID-19 testing) may be an over or underestimate of the true excess mortality of COVID-19, due to the representativeness of COVID-19 as the true underlying or contributory cause of death. Finally, we were unable to derive confidence intervals at each stage and in aggregate due to constraints on computational resources. We understand that the application of COVID-19 mortality displacement to adjust conventional expected deaths models and the concept of excess mortality may be undertaken using different methods and further investigative work is required.

As in any model-based analysis, these limitations should be considered in interpreting results. However, compared with other complex modelling scenarios the data we have used are informationally very rich and should undoubtedly provide a useful approximation to what is happening in reality. Furthermore, if one focuses on the method—as a way to build and use a platform to provide information that may be used to help guide public health intervention in a wide variety of ways during and following on from a substantial population-level public health shock—the limitations we highlight provide useful guidelines for how such platforms should be set up and evolve.

### Implications and conclusions

Directly observing individuals within a population over time, monitoring events of interest and applying the methodology we have described has applications for answering a multitude of potential public health questions. We describe and apply the approach to temporal displacement of all-cause mortality. But it can equally well be applied to the displacement of cause-specific death and to adjustment of excess mortality associated with specific causes. This will allow better understanding of longer-term impacts of COVID-19 on important causes of death. The methods can also be extended to any adverse outcome (death is simply one example) following any ‘at-risk’ defining event (the example here being developing COVID-19). Furthermore, by assuming everybody in the population is ‘at risk’ from the moment a pre-defined event occurs, death (or another metric) can be tracked as the primary outcome. In all situations such as these, which include extreme temperature events, it is possible to estimate the long-term impact of any population-level event on any outcome – thereby providing critical information for public health planning.

Throughout the pandemic it has become increasingly apparent that there is a worldwide need to link individual-level datasets from various sources to create population-level platforms. Setting up country-wide cohorts starting at prespecified times and recording critical events of interest, could provide critical information for the purposes of public health surveillance, planning and intervention. Using data from such platforms, our work demonstrates that by applying the extended Cox model and subsequently simulating the expected outcomes in both factual and counterfactual scenarios it would be possible to answer a multitude of questions (e.g., at this time, the impact of COVID-19 infection on long-term comorbidity, health service utilisation and other health outcomes). The benefits of having a population platform such as this, and applying analytic methods, including modelling, at the individual-level offers numerous benefits. Using whole populations with individual-level data has the benefit of greater precision and richer mathematical models which provide a more robust approach to addressing the real challenges that are faced by public health science as well as nations themselves. We believe that the ability to generate critical information to inform policy decisions and health planning, as well as help manage major public health shocks, would make an investment in an infrastructure based on pseudonymised data to track real-time, pan-population health events worthwhile given that the scientific and social returns that greatly exceed its costs.

### Supplementary Information

**Additional file 1: Supplementary 1 Material A.** Fitting the Cox proportional hazard model. **Supplementary Material B.** Simulation protocol. **Supplementary Material C.** Comparison of the simulated actual and counterfactual scenarios. **Supplementary Material D.** Extrapolation of the Kaplan Meier survival curve. **Supplementary Material E.** Applying the displacement correction to excess mortality estimates.

## Data Availability

Secondary sharing of the study data is not permitted, however the underlying data that support this study are available through formal application to the Falls and Fragility Fracture Audit Programme and UK Healthcare Quality Improvement Partnership Data Access Request Group. Data concerning excess mortality in England on which this study is based is published monthly by OHID and available publicly at https://www.gov.uk/government/statistics/excess-mortality-in-england-and-english-regions.

## References

[1] Office for National Statistics. Deaths registered weekly in England and Wales, provisional. https://www.ons.gov.uk/peoplepopulationandcommunity/birthsdeathsandmarriages/deaths/bulletins/deathsregisteredweeklyinenglandandwalesprovisional/weekending11march2022. Accessed 12 Apr 2023.

[2] Scottish Government. COVID-19 detailed analysis. https://data.gov.scot/coronavirus-covid-19/detail.html. Accessed 12 Apr 2023.

[3] Office for Health Improvement and Disparities. Excess mortality in England and English regions. GOV.UK. 2023. https://www.gov.uk/government/statistics/excess-mortality-in-england-and-english-regions. Accessed 31 Mar 2022.

[4] Eurostat. Excess mortality - statistics. https://ec.europa.eu/eurostat/statistics-explained/index.php?title=Excess_mortality_-_statistics. Accessed 12 Apr 2023.

[5] National Center for Health Statistics. Excess deaths associated with COVID-19. 2023. https://www.cdc.gov/nchs/nvss/vsrr/covid19/excess_deaths.htm. Accessed 12 Apr 2023.

[6] Barnard S, Chiavenna C, Fox S, Charlett A, Waller Z, Andrews N (2022). Methods for modelling excess mortality across England during the COVID-19 pandemic. Stat Methods Med Res.

[7] Xie Y, Xu E, Bowe B, Al-Aly Z (2022). Long-term cardiovascular outcomes of COVID-19. Nat Med.

[8] Office for National Statistics. Excess mortality and mortality displacement in England and Wales - Office for National Statistics. https://www.ons.gov.uk/peoplepopulationandcommunity/birthsdeathsandmarriages/deaths/articles/excessmortalityandmortalitydisplacementinenglandandwales/2020tomid2021. Accessed 18 Mar 2023.

[9] Rehman H, Chandra N, Jammalamadaka SR (2021). Competing risks survival data under middle censoring—An application to COVID-19 pandemic. Healthcare Analytics.

[10] Bhaskaran K, Bacon S, Evans SJ, Bates CJ, Rentsch CT, MacKenna B (2021). Factors associated with deaths due to COVID-19 versus other causes: population-based cohort analysis of UK primary care data and linked national death registrations within the OpenSAFELY platform. Lancet Reg Health Eur.

[11] Department of Health and Social Care. Additional £5.4 billion for NHS COVID-19 response over next 6 months. GOV.UK. https://www.gov.uk/government/news/additional-54-billion-for-nhs-covid-19-response-over-next-six-months. Accessed 12 Apr 2023.

[12] NHS Digital. Compendium - Deaths within 30 days of a hospital procedures or emergency admission. NDRS. https://digital.nhs.uk/data-and-information/publications/statistical/compendium-hospital-care/current/deaths-within-30-days. Accessed 12 Apr 2023.

[13] National Institute for Health and Care Excellence (2020). Perioperative care in adults [C] Evidence review for preoperative risk stratification tools NICE guideline NG180.

[14] Bottle A, Aylin P, Warner M, Propper C, Stoye G, Burn S (2021). What happened to English NHS hospital activity during the COVID-19 pandemic?.

[15] British Medical Association. NHS backlog data analysis. The British Medical Association is the trade union and professional body for doctors in the UK. https://www.bma.org.uk/advice-and-support/nhs-delivery-and-workforce/pressures/nhs-backlog-data-analysis. Accessed 12 Apr 2023.

[16] Huynen MM, Martens P, Schram D, Weijenberg MP, Kunst AE (2001). The impact of heat waves and cold spells on mortality rates in the Dutch population. Environ Health Perspect.

[17] Grize L, Huss A, Thommen O, Schindler C, Braun-Fahrländer C (2005). Heat wave 2003 and mortality in Switzerland. Swiss Med Wkly.

[18] Royal College of Physicians, London. National Hip Fracture Database (NHFD). RCP London; 2015. https://www.rcplondon.ac.uk/projects/national-hip-fracture-database-nhfd. Accessed 12 Apr 2023.

[19] Therneau T, Crowson C, Atkinson E. Using time dependent covariates and time dependent coe cients in the cox model. A vignette for the survival package in R. https://cran.r-project.org/web/packages/survival/vignettes/timedep.pdf.

[20] Holleyman RJ, Khan SK, Charlett A, Inman DS, Johansen A, Brown C (2022). The impact of COVID-19 on mortality after hip fracture: a population cohort study from England. Bone Joint J.

[21] Cox DR (1972). Regression models and life-tables. J Roy Stat Soc Ser B (Methodol).

[22] Breslow N (1972). Discussion of regression models and life-tables by Cox et al. J Roy Statist Assoc B.

[23] NHS Digital. Hospital Episode Statistics (HES). NHS Digital. https://digital.nhs.uk/data-and-information/data-tools-and-services/data-services/hospital-episode-statistics. Accessed 8 Nov 2022.

[24] Office for National Statistics. Office for National Statistics - Registered deaths. https://www.ons.gov.uk/peoplepopulationandcommunity/birthsdeathsandmarriages/deaths. Accessed 27 Mar 2023.

[25] UK Government. About the data - coronavirus (COVID-19) in the UK. https://coronavirus.data.gov.uk. Accessed 6 Sep 2022.

[26] Armitage P, Berry G, Matthews JN. Statistical methods in medical research. John Wiley & Sons; 2008.

[27] Kaplan EL, Meier P (1958). Nonparametric estimation from incomplete observations. J Am Stat Assoc.

[28] NHS Digital. Deaths within 30 days of emergency admission to hospital: fractured proximal femur: indirectly standardised rate, all ages, annual trend, F,M,P. NHS Digital. https://digital.nhs.uk/data-and-information/publications/statistical/compendium-hospital-care/current/deaths-within-30-days/deaths-within-30-days-of-emergency-admission-to-hospital-fractured-proximal-femur-indirectly-standardised-rate-all-ages-annual-trend-f-m-p. Accessed 12 Apr 2023.

[29] Devleesschauwer B, McDonald SA, Speybroeck N, Wyper GMA (2020). Valuing the years of life lost due to COVID-19: the differences and pitfalls. Int J Public Health.

[30] Hanlon P, Chadwick F, Shah A, Wood R, Minton J, McCartney G (2020). COVID-19 - exploring the implications of long-term condition type and extent of multimorbidity on years of life lost: a modelling study. Wellcome Open Res.

[31] Pifarré I, Arolas H, Acosta E, López-Casasnovas G, Lo A, Nicodemo C, Riffe T (2021). Years of life lost to COVID-19 in 81 countries. Sci Rep.

[32] Quast T, Andel R, Gregory S, Storch EA (2022). Years of life lost associated with COVID-19 deaths in the USA during the first year of the pandemic. J Public Health (Oxf).

[33] Austin PC (2012). Generating survival times to simulate Cox proportional hazards models with time-varying covariates. Stat Med.

[34] Cheng J, Xu Z, Bambrick H, Su H, Tong S, Hu W (2018). Heatwave and elderly mortality: an evaluation of death burden and health costs considering short-term mortality displacement. Environ Int.

[35] Rivera R, Rosenbaum JE, Quispe W (2020). Excess mortality in the United States during the first three months of the COVID-19 pandemic. Epidemiol Infect.

[36] Alicandro G, Remuzzi G, La Vecchia C (2020). Italy’s first wave of the COVID-19 pandemic has ended: no excess mortality in May, 2020. Lancet.

[37] Michelozzi P, de’Donato F, Scortichini M, Pezzotti P, Stafoggia M, De Sario M (2020). Temporal dynamics in total excess mortality and COVID-19 deaths in Italian cities. BMC Public Health.

[38] Canouï-Poitrine F, Rachas A, Thomas M, Carcaillon-Bentata L, Fontaine R, Gavazzi G (2021). Magnitude, change over time, demographic characteristics and geographic distribution of excess deaths among nursing home residents during the first wave of COVID-19 in France: a nationwide cohort study. Age Ageing.

[39] Scortichini M, Schneider Dos Santos R, De’ Donato F, De Sario M, Michelozzi P, Davoli M (2021). Excess mortality during the COVID-19 outbreak in Italy: a two-stage interrupted time-series analysis. Int J Epidemiol.

[40] GBD 2019 Diseases and Injuries Collaborators (2020). Global burden of 369 diseases and injuries in 204 countries and territories, 1990–2019: a systematic analysis for the Global Burden of Disease Study 2019. Lancet.

[41] Rypdal M, Rypdal K, Løvsletten O, Sørbye SH, Ytterstad E, Bianchi FM (2021). Estimation of excess mortality and years of life lost to COVID-19 in Norway and Sweden between March and November 2020. Int J Environ Res Public Health.

[42] Wyper GMA, Fletcher E, Grant I, McCartney G, Fischbacher C, Harding O (2022). Measuring disability-adjusted life years (DALYs) due to COVID-19 in Scotland, 2020. Arch Public Health.

[43] Andrasfay T, Goldman N (2021). Reductions in 2020 US life expectancy due to COVID-19 and the disproportionate impact on the Black and Latino populations. Proc Natl Acad Sci U S A.

[44] Rozenfeld Y, Beam J, Maier H, Haggerson W, Boudreau K, Carlson J (2020). A model of disparities: risk factors associated with COVID-19 infection. Int J Equity Health.

[45] Pijls BG, Jolani S, Atherley A, Derckx RT, Dijkstra JIR, Franssen GHL (2021). Demographic risk factors for COVID-19 infection, severity, ICU admission and death: a meta-analysis of 59 studies. BMJ Open.

