# Supplementary material for: Adjusting expected deaths for mortality displacement during the COVID-19 pandemic: a model based counterfactual approach at the level of individuals
Source: BMC Med Res Methodol. 2023 Oct 18;23:241. doi: 10.1186/s12874-023-01984-8 (PMC10585864; doi:10.1186/s12874-023-01984-8)
Supplement: Supplementary file 1 — Additional file 1: Supplementary 1 Material A. Fitting the Cox proportional hazard model. Supplementary Material B. Simulation protocol. Supplementary Material C. Comparison of the simulated actual and counterfactual scenarios. Supplementary Material D. Extrapolation of the Kaplan Meier survival curve. Supplementary Material E. Applying the displacement correction to excess mortality estimates. [file 12874_2023_1984_MOESM1_ESM.pdf]

Supplementary Material A

Fitting the Cox proportional hazard model

```
cox.model<-coxph(survmod~age60+female+bmigp.1+bmigp.2+diab +
        tt(COVID_19.date.1) + tt(COVID_19.date.2)+
        tt(COVID_19.date.3) + tt(COVID_19.date.4),
        x=TRUE,ties="breslow",
        tt=list(
          function(x,t, ...)
                {
                week1.post.COVID_19<-((t>=x)& t<(x+7))
                },
          function(x,t, ...)
                {
                week2.post.COVID_19<-((t>=x+7)& t<(x+14))
                },
        function(x,t, ...)
                {
                week3.post.COVID_19<-((t>=x+14)& t<(x+21))
                },
          function(x,t, ...)
                {
                week4.post.COVID_19<-((t>=x+21)& t<(x+84))
          } ))
```

Box 1: the type of Cox model typically required for our approach

Box 1 outlines a typical Cox model of the type that provides the foundation for the analyses described in this document. Model fitting is in R using the coxph(.) function. Model construction starts with the used of the Surv(.) function that converts the raw survival times and censoring statuses from the original data set into a Surv object (here called survmod): survmod<-Surv(survival.time,failed). The R object ‘survmod’ can now be declared as the outcome term of a Cox model fitted using coxph (*i.e.* the first argument in the regression formula). The next few terms in the formula typically represent a set of time fixed covariates that are felt to appropriately capture at least some of the key variability in the risk of death in the absence of COVID-19. Here, for illustrative purposes, these are age, sex, BMI, and presence/absence of diabetes. Covariates designated “tt(*variable name*)” are time dependent covariates where the time dependency in each case is defined by a function (declared in the tt list that follows) that determines the value of that covariate in every risk set (*i.e.* at every unique failure time). Within each function, x specifies the variable identified as *variable name* in the original call to tt(.) and t is the time corresponding to whatever risk set is being considered. The functions in the tt list are always in the same order as the corresponding tt() covariates in the model specification. For this illustration using exemplar code, t is measured in days since the day an individual entered follow-up (t = 0) which is itself defined as the day that individual had a hip fracture recorded in the national hip fracture registry. In the real analysis we later changed the primary analytic time-scale from days to weeks to reduce computing load. The variables, COVID_19.date.1 … COVID_19.date.4 are all copies of an original variable COVID_19.date that specifies the date (if any) at which a positive COVID-19 test was reported in each individual – the date being specified in days on the same scale (t) as the basis of the primary analysis (*i.e.* 0 refers to the start of follow-up for any given individual). Anyone who *never* had a positive COVID-19 test is allocated an arbitrary, but large, value for COVID_19.date (and therefore all four of the copy variables) that is greater than the maximum follow-up time of any subject in the data set. The need to declare four exact copies of the same variable is a quirk of the coxph(.) function which does not allow the same variable name to be used for more than one tt covariate.

As an illustration, and given these definitions, the covariate week2.post.COVID_19 will take the value 1 in any risk set where the date of the risk set (t) is greater than or equal to 7 days after the t corresponding to the first positive COVID-19 test in a particular individual and less than 14 days after that same positive test in that individual. At all other times it takes the value 0. This defines a time-dependent covariate that is a binary step function (either 1 or 0

on any given date) and its coefficient will reflect the risk of death in the second week after a positive COVID-19 test relative to all other times.

All covariates and their standard errors are estimated directly from the model fit above. But estimating the baseline hazard, which is also crucial, is slightly more difficult than normal. This is because the function that is normally used to generate the baseline hazard function in R – that is, basehaz(.) – has not been generalized to models containing time-dependent covariates. In consequence, because our approach demands an estimate of the baseline hazard function *and* requires that we use time dependent covariates we estimated the baseline hazard at each failure time using the method described by Breslow [24] in the discussion of the original paper describing the Cox model [49].

Supplementary Material B

Simulation protocol

The basis for the simulation in the actual and counterfactual scenarios is summarised by the following protocol:

(1) Start simulation 1 of M.
(2) Start at time 0 with all (N) subjects alive (*i.e.* censoring status = 0 for everybody).
(3) Consider subject i = 1 at unique failure time u = 1. Use the Cox model results and Equation 1 (main manuscript) to estimate $\lambda(1)_1$, the instantaneous combined hazard for subject 1 at unique failure time u = 1. A unique failure time refers to a point in time on the primary time scale of the Cox model when at least one person died – that is, it defines a risk set in the Cox model. In general, we often abbreviate the term "unique failure time" to uft but when, in the following description of the simulation algorithm, we are referring to the time at which a particular uft occurred, we shorten this to u to save space and make the text easier to read.
(4) Use equation $f(t)_i = 1 - e^{-\lambda(t)_i}$ [22, 24] (see main manuscript, Methods section 2) to transform $\lambda(1)_1$ to $f(1)_1$, the probability that subject 1 dies at u = 1 given that they are alive arriving at u =1.
(5) In R, use the function rbinom(1,1, $f(1)_1$) to generate a random Bernoulli (0,1) value $\mathcal{B}(1)_1$, where $f(1)_1$ is the probability that $\mathcal{B}(1)_1$ is 1 and $1 - f(1)_1$the probability it is 0.
(6) If $\mathcal{B}(1)_1$ = 1, declare that subject 1 died at u = 1 and that the simulated outcome data in subject 1 is complete: survival time = 1; censoring status = 1 (*i.e.* died at time = 1). This terminates the data synthesis for subject 1 in iteration 1. Return to step 3 moving on to subject i = 2, 3, 4 … N and keep repeating steps 3-8.
(7) If $\mathcal{B}(1)_1$ = 0 declare that subject 1 survived through u = 1, return to step 3 and keep repeating steps 3-7 for u = 2, 3, 4, …. u_{max} until reaching uft u at which $\mathcal{B}(u)_1 = 1$. Then declare that the simulated outcome data in subject 1 is complete: survival time = u; censoring status = 1 (*i.e.* died at time = u). This completes the data synthesis for subject 1 in iteration 1. Return to step 3 moving on to subject i = 2, 3, 4 … N and keep repeating steps 3-8
(8) If $\mathcal{B}(u)_1 = 0$ at the end of observed follow-up *i.e.* at u = u_{max} declare that the simulated outcome data in subject 1 is complete: survival time = u_{max}; censoring status = 0 (*i.e.* still alive at time = u_{max}). This completes the data synthesis for subject 1 in iteration 1. Return to step 3 moving on to subject i = 2, 3, 4 … N and keep repeating steps 3-8
(9) When the current simulation is complete for subject N declare that that simulation is closed and save the results as two vectors of N survival times and N values for censoring status. Return to step 2 and repeat all steps for simulations 2, 3, 4 … M

(10) When simulation M is complete, save all results from all simulations as two matrices each with N rows and M columns where element n,m in $matrix_{survtime}$ represents the survival time of subject n in simulation m and element n,m in $matrix_{censor}$ holds the corresponding censoring status. The simulation procedure is now complete.

If any given individual has no failures is *any* of the simulations, they get recorded as censored at u_{max} in every simulation. In effect, this means we know with high certainty that they should survive beyond the period covered by our analysis of mortality displacement. But there is almost no information about *how long* they may be expected to survive beyond this period. This issue is discussed in detail in *Discussion* in considering the differences between analysing mortality displacement and years of life lost (YLL) and when, and when not, our method may sensibly be used.

Supplementary Material C

Comparison of the simulated actual and counterfactual scenarios

For the first application of the simulation protocol, simulating the *actual* (real world) scenario, the distribution of the simulated dates of death in each subject across the M simulations should approximately reflect the observed time of death in that same individual in the original real data. However, that reflection should, in principle, be systematically biased. The reason for this is well illustrated by someone who dies very early (*e.g.* on the second uft). In most simulations they will, by chance be simulated as having a later date of death than was observed (simply because it is only if they are simulated as dying at the first uft that their simulated value can be lower than their observed value). If they are simulated as dying at the second uft the observed and simulated values will be equal. But, if they die at *any other* uft their simulated value will exceed their observed value. However, perhaps non-intuitively, this 'bias' is necessary. This is because, based on an argument closely related to regression to the mean and caused directly by random chance, those individuals who actually died closest to the start of follow-up will often have died earlier (but rarely later) than their real survival time expected given their covariate pattern. It is therefore appropriate that under stochastic simulation their simulated survival times will more often exceed their observed survival times than occur earlier. Analogously, the opposite bias will occur in individuals observed to have particularly long survival times. Crucially, this means that when we come to compare survival times under the actual and counterfactual scenarios, we *must* use the distribution of the *simulated* survival times for everybody under the real world scenario even though it may appear intuitively more appealing to use their *observed survival times*.

For the second application, simulating the counterfactual scenario, the baseline hazard remains precisely the same as under the previous scenario. This is because, in the full model, the impact of COVID-19 is modelled entirely by the COVID-19 covariates and this implies that the baseline hazard - defined as being the hazard *when all covariates are set to zero* - will appropriately model the hazard in someone who does not have COVID-19 regardless whether that is because they haven't recently been infected by COVID-19 (actual scenario), they never develop COVID-19, or because we are assuming *nobody* is infected by COVID-19 (*i.e.* the counterfactual scenario).

To avoid confusion, it should be noted that the baseline hazard at any time t applies to *everybody* who is still alive at time t (regardless whether or not they have COVID-19 at that time or at any other time). Crucially, the baseline hazard does *not* represent the *'underlying risk of death amongst people without COVID-19'* which it may intuitively be misinterpreted to represent. In other words, by modelling the data in the way we are, the baseline hazard reflects the instantaneous risk of death from all causes in *anybody* (for convenience in

scaling, being estimated as the instantaneous hazard for someone in whom all covariates – including the COVID_19 covariates – are all zero). The extent to which this risk is then increased when someone is actively infected with COVID-19 is modelled entirely by the four time dependent COVID_19 covariates. This parameterisation allows us to completely disentangle that enhanced risk associated with COVID-19 infection from the risks shared by everybody (*i.e.* accounted for by the baseline hazard and all non-COVID_19 covariates).

Given this theory, once the M simulations for the actual scenario are complete and the expected survival profiles of everybody in the study population have been estimated, the same process can now be repeated on the same study population, but under the counterfactual scenario: that is, using the modified covariate structure with the COVID-19 covariates set to zero for all individuals at all times. Among subjects that did not contract COVID-19 the two sets of simulations should generate to very close approximation the same expected times of death (and corresponding survival times) under both models. Any minor variation that *is* observed is consequent solely on stochastic variation in the simulations. In contrast, among those that did test positive to COVID-19, the probability of death under the counterfactual scenario during the period when, in reality, a subject actually had COVID-19, will be markedly reduced because of the removal of the effect of the COVID-19 covariates.

Supplementary Material D

Extrapolation of the Kaplan Meier survival curve

Using the approach outlined above, a key challenge is how to estimate the expected median survival time (*i.e.* the follow-up time at which it is estimated precisely half of simulations will have failed) for individuals in whom, in actuality, less than 50% of simulations have failed even though the maximum follow-up time has been achieved. This requires extrapolation of the Kaplan Meier survival function beyond the observed simulated data. As our work progressed, we recognised an evolving range of general approaches and technical details that could enhance such extrapolation.

First, if instead of following our study population from close to the start of the COVID-19 pandemic, we instead followed them from say, a year earlier, our total follow-up time and number of ufts could be markedly increased. This reduced the proportion of individuals whose simulations failed to reach a 50% probability of dying before the end of the simulations. It may appear counterintuitive that increasing the follow-up time over periods when nobody had COVID-19 could refine our estimates of the impact of COVID. However, it is in fact logical. First, it increased the observed number of deaths in low risk subjects (including those without COVID), and therefore increased statistical power. Second, as we see below, a particular extrapolation problem can arise in people who develop COVID-19 close to the end of the maximum follow-up time. By starting follow-up substantially before the COVID-19 pandemic started, the only way that the COVID-19 covariates could impact on simulations at the ufts close to the maximum follow-up times would be in individuals who were *both* recruited early (*i.e.* had a hip fracture substantially ahead of 2020) and were tested for COVID-19 very close to the end of follow-up *e.g.* in April 2021. Inevitably, there were relatively few of these.

Second, we began by arguing that it was primarily individuals who tested positive for COVID-19 and *also* died that could be responsible for mortality displacement and so only compared the two survival time distributions in these individuals. But, the distribution of simulated survival times in the COVID-19 scenario was substantially positively biased relative to the observed survival times in this group. We ultimately recognised that this was consequent upon the issue, closely related to regression to the mean, that was addressed in

Supplementary Material C. Specifically, if an individual in reality, and entirely by chance, died earlier than might have been expected from their risk profile they *could* appear in the sample of people who died and had COVID. In contrast, if an individual died unexpectedly late, again entirely by chance, they may well be missing from this analytic sample because they didn't actually die during the follow-up period. However, because the simulated survival times based on observed covariate patterns are not biased in this way (see Supplementary Material C), the distribution of observed survival times in the actual sample would inevitably appear, on average, shorter than the unbiased simulated survival times.

Before we realised that the bias was in the observed survival times rather than their simulated equivalents, we set out to correct the simulated estimates, so they were less biased relative to the observed estimates. As an initial solution, we excluded anybody from the sample who actually died before the end of follow-up if their median *simulated survival time* under the COVID-19 scenario was greater than the total follow-up time. This did indeed mitigate the bias between the observed and simulated survival times. But we later realised this was because we were imposing a bias on the simulated data that was equivalent to that in the observed data. In addition, we recognised that we were discarding people who were informative for our analysis. In consequence, once we properly understood the nature of the bias and realised that it applied to the observed survival times rather than the simulated survival times, we determined that the comparative analysis ought to include *anybody* who had tested positive for COVID-19 regardless whether they died or not and whether or not their median simulated survival time was less than the total follow-up or not. Finally, to echo Supplementary Material C, we also realised that it was their *simulated* expected median survival times in the actual (real world) scenario that should be compared to their simulated equivalents under the counterfactual scenario: not their *observed* survival times in the real world data.

This latter approach successfully circumvented much of the bias that we had previously seen. However, we still needed to construct a valid estimate of the expected median survival time in individuals in whom less than 50% of the simulations had failed before the end of follow-up. To do this we argued that if the underlying risk of death remains precisely constant over time, the survival curve would follow an exponential survival distribution – the archetype for radioactive decay. In that case it is well known that the half-life is constant and that the survival function (the probability of survival over linear time) itself curves with a gradually flattening gradient. This means any direct extrapolation of the survival function itself must take proper account of curvature. But, a plot of the logarithm of the probability of survival against linear time is a straight line. Furthermore, the log of the probability of survival is equal to the negative cumulative hazard. In consequence, in order to extrapolate any Kaplan Meier survival function that failed to fall as low as 50% we chose to extrapolate the (rising) straight line representing the cumulative hazard versus time and used this to identify the future date at which the cumulative hazard would be expected to attain the value $\log_e 2$ *i.e.* 0.6931. This corresponds to a survival probability of $\exp(-\log_e 2)$ which equals one half and therefore corresponds to 50% survival (*i.e.* the expected median survival time).

A final piece of theory that applies to the method of extrapolation is that, in real data, the true curve of survival probability over linear time does not precisely represent an exponential decay. This is because truly exponential decay demands that the risk of failure of each 'individual' is constant over time. In the specific case of our simulations, this would imply that the risk of death at every uft for any given individual is always the same. But, in reality the risk of death varies across ufts because of changes in the baseline hazard and/or because of time dependent covariates. However, in practice, the empirical plots of cumulative hazard against linear time were typically quite close to straight lines *except* during the period when an individual was sick with covid. During this period, when the time-dependent covariates modelling the impact of COVID-19 were being switched on and off sequentially, the approximate straight line increase of the cumulative hazard over time markedly increased in gradient and this meant that the overall relationship between cumulative hazard and time,

from the start to the end of follow-up time would be far from linear. For this reason, when extrapolation was required, we used only the last 25% of the cumulative hazard v survival time graph as the basis for extrapolation. In the vast majority of subjects, this was based on simulated outcomes *after* COVID-19 had been and gone and the extrapolation was unbiased. Crucially, extrapolation of the cumulative hazard versus survival time curve in the counterfactual (NO COVID) scenario is not disturbed by the COVID-19 covariates because they are set to zero at all times. Finally, it might be argued that we should have used a more sophisticated extrapolation approach – *e.g.* based on splines to account for curvature. However we were concerned that this could result in overfitting with an enhanced fit to the observed data in any given individual but generating wildly diverging extrapolation lines in some individuals once one moved beyond the observed data. In addition, we were able to demonstrate good consistency in predicted median survival times based on our simple method even if, for example, we changed from using the last 25% of the curve to the last 33% or last 50%.

Supplementary Material E

Applying the displacement correction to excess mortality estimates

There are three important issues to note: (1) The area under the curve representing the distribution of survival time shifts sums to 1.00 – so the total number of balls removed from future urns each week is precisely equal to the number of deaths in people with COVID-19 in the week being accounted for. However, some of these could in theory be removed from urns that are years into the future and beyond the scope of the expected deaths at present. (2) For convenience, the analogy of balls in urns is used, but in reality, expected deaths are not necessarily integers, and neither are the changes made to the expected deaths by this method. (3) Zero and negative time shifts are both mathematically possible and theoretically plausible. They arise if a death occurs after COVID-19 has been diagnosed but the death either occurs on the same date that the subject would have died anyway, or else the death occurs before it would otherwise have done. Zero time shifts appear quite reasonable but COVID-19 delaying the date of death may seem counter-intuitive. But one real world scenario where this could happen would be if someone would otherwise have died during an elective procedure that was cancelled because of COVID. As well as being theoretically plausible, such shifts are an inevitable feature of the stochastic method by which the simulated survival data are generated and so when Δ_j contains negative values the adjustment procedure remains consistent, we simply remove a small number of balls from urns representing earlier weeks. In reality, negative shifts are only rarely predicted.
